# Stepwise error-prone PCR and DNA shuffling changed the pH activity range and product specificity of the cyclodextrin glucanotransferase from an alkaliphilic *Bacillus* sp.

**DOI:** 10.1016/j.fob.2015.06.002

**Published:** 2015-06-11

**Authors:** Susanne Melzer, Christian Sonnendecker, Christina Föllner, Wolfgang Zimmermann

**Affiliations:** Institute of Biochemistry, Department of Microbiology and Bioprocess Technology, Leipzig University, Johannisallee 23, 04103 Leipzig, Germany

**Keywords:** CD, cyclodextrin, CGTase, cyclodextrin glucanotransferase, Cyclodextrin glucanotransferase, *Bacillus* sp., Gamma-cyclodextrin, Random mutagenesis, DNA shuffling

## Abstract

Cyclodextrin glucanotransferase (EC 2.4.1.19) from the alkaliphilic *Bacillus* sp. G-825-6 converts starch mainly to γ-cyclodextrin (CD_8_). A combination of error-prone PCR and DNA shuffling was used to obtain variants of this enzyme with higher product specificity for CD_8_ and a broad pH activity range. The variant S54 with seven amino acid substitutions showed a 1.2-fold increase in CD_8_-synthesizing activity and the product ratio of CD_7_:CD_8_ was shifted to 1:7 compared to 1:3 of the wild-type enzyme. Nine amino acid substitutions of the cyclodextrin glucanotransferase were performed to generate the variant S35 active in a pH range 4.0–10.0. Compared to the wild-type enzyme which is inactive below pH 6.0, S35 retained 70% of its CD_8_-synthesizing activity at pH 4.0.

## Highlights

• We performed random mutagenesis experiments with a cyclodextrin glucanotransferase.
• Error-prone PCR and DNA shuffling steps were combined.
• Variants with a broad pH activity range could be obtained.
• Several variants showed increased product specificity for γ-cyclodextrin.

## Graphical abstract

**Figure:**
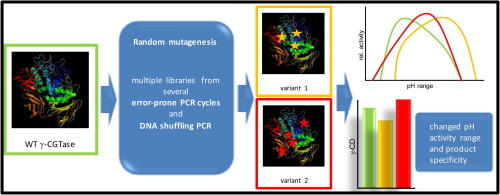

## 1 Introduction

Cyclodextrins (CD) are cyclic molecules composed of 6+n α-1,4 -linked glucose residues. CD_6_, CD_7_ and CD_8_, consisting of 6, 7 and 8 glucose residues, are commercially produced and are also designated as α-CD, β-CD and γ-CD [1]. The hydrophobic cavity of CD allows the formation of inclusion complexes with guest molecules with various applications e.g. in the food [2] and pharmaceutical industries [2–5]. CD are synthesized by cyclodextrin glucanotransferases (CGTases, EC 2.4.1.19) from starch [6]. They have been classified as α-CGTase, β-CGTase and γ-CGTase according to the main CD product formed. However, all known CGTases produce a mixture of CD of different sizes requiring costly and time-consuming separation steps to obtain single CD of a specific size required for many applications [7]. Therefore, CGTases forming CD of only one size are desirable for industrial CD production processes.

A comparison of the temperature- and pH-optima of various CGTases has indicated that most α-CGTases showed their highest activity at low pH conditions and at higher temperatures [8,9], while β-CGTases displayed their optimum activity at low to neutral pH over a wide temperature range [10–13]. In contrast, γ-CGTases, frequently detected in alkaliphilic bacteria, were prseferentially active at high pH conditions [14–18].

The monomeric CGTases are composed of five domains (A to E) [19]. The A domain forms a (β/α)-8 barrel structure. The B domain consists of a loop between β-sheet 3 and α-helix 3 of the A domain. Close to the active center, secondary carbohydrate binding sites (subsites) have been identified [19,20].The catalytic site and the subsites are located within the A/B domains. The domains C and E contain starch binding sites, whereas the function of domain D has not been fully elucidated [21,22]. The catalytic triad consisting of two Asp and a Glu residue is localized in the domain A and is highly conserved in all α-amylases. The catalytic center forms a deep groove to allow an interaction with oligo- and polysaccharide substrates [20].

Protein engineering of CGTases has been performed previously to improve their substrate and product specificity, as well as their thermostability [23]. By site-directed mutagenesis, the product specificity of α- [24–26], β- [27,28] and γ-CGTases [29–31] has been enhanced successfully. Replacement of amino-acids at the subsite −3 [25,27,31], −6 [24], and −7 [26] resulted in changes of the CD product spectrum and enzyme activity. By site-directed mutagenesis, a CGTase variant with enhanced thermostability has been obtained by introduction of an additional salt bridge [32]. However, an improvement of the pH activity range of CGTases by protein engineering has not been reported yet.

In this study the modification of the pH activity range and product specificity of a γCGTase using a stepwise random mutagenesis strategy is described.

## 2 Results and discussion

### 2.1 Mutagenesis and screening of cgtS shuffling variants

**Fig. 1:**
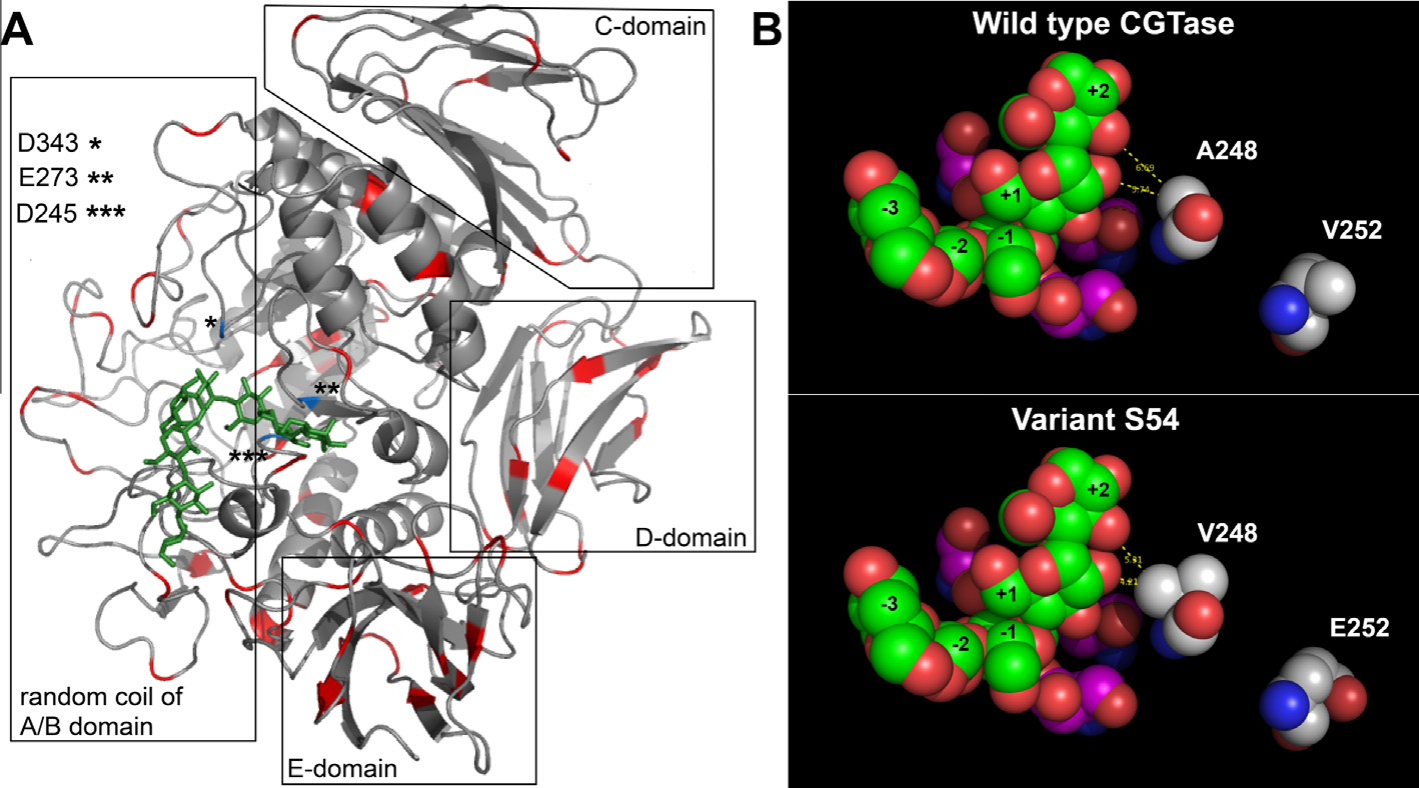
Structure comparison of the wild-type γ-CGTase G825-6 (A) and the variant S54 (B). Ribbon diagram of the wild-type γ-CGTase G825-6 (A). The location of the mutations in all variants obtained by random mutagenesis are colored in red. Comparison of the sphere model of the active site of the variant S54 with the amino acid substitutions A248V and V252E and the wild-type γ-CGTase G825-6 (B). C-atoms of amino acid 248 and 252 are highlighted in grey. The active site residues D245, E273 and D343 are colored in pink, the maltononaose substrate in green, O atoms in red and N atoms in blue. Distances are indicated with yellow dashed lines. The models were generated using SWISS-MODEL with *1cygA* (PDB) as template structure [68] <http://www.sciencedirect.com/science/article/pii/S221154631500056X#b0340>. The maltononaose substrate (1CXK PDB) was superimposed by structure alignment. Visualization was performed with PyMol V0.99.

By repeated rounds of low-frequency mutations (two error-prone PCR followed by DNA shuffling) with repeated selection, CGTase variants with increased CD_8_-synthesizing activity in comparison to the wild-type enzyme could be identified. Using high-mutagenic conditions, only non-functional variants were obtained. The created mutations were evenly distributed within the CGTase sequence (Fig. 1A, Table 1). By stepwise random mutagenesis of the *cgtS* gene, 15.000 clones were obtained and subsequently screened for CD_8_-synthesizing activity on congored agar plates containing 1% soluble starch. Congored is a secondary diazo dye soluble in water and red colored at pH 5.2 and above. In the presence of γ-CGTase, CD_8_ is produced by the conversion of starch. The dye forms a complex with CD_8_ and becomes colorless resulting in a halo around the colony on the plate [33]. The assay is highly selective for CD_8_, since no halos were formed with CD_6_, CD_7_, CD_9_, CD_10_ or a mixture of large ring CD (CD_22_–CD*_n_*). More than 50 halo-producing clones were detected, 21 of these with the largest halo areas were used for further analysis of their CD_8_-synthesizing activity and specificity.

### 2.2 pH activity range of the CGTase variants

**Fig. 2:**
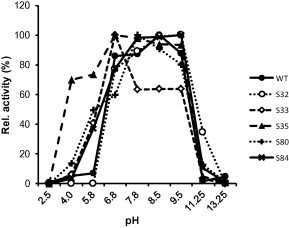
pH activity range of the wild-type γ-CGTase G825-6 and the variants S32, S33, S35, S80 and S84. The maximum amount of CD_8_ produced by each of the variants at their pH optimum was set to 100%.

While CD_8_-synthesizing activity of the wild-type γ-CGTase was detected between pH 6.8 and 9.5, five of the 21 analyzed shuffling variants (S32, S33, S35, S80 and S84) showed activity over a broader pH range (Fig.2). The variants S80 and S35 (active between pH 4.0 and 9.5) retained 14% and 70% of their CD_8_-synthesizing activity at pH 4.0, respectively. The variants S32 (active between pH 6.8 and 11.25) and S84 retained 34% and 10% of their activity at pH 11.25, respectively. The variant S33 retained even 65% of its activity at pH 5.8. By stepwise random mutagenesis, a γ-CGTase could be created with CD_8_-synthesizing activity at low pH as a novel and unusual property of the enzyme. For an industrial production of CD from starch pretreated with an α-amylase at pH 6.0 to 6.5, a pH adjustment step could thus be eliminated by employing a CGTase active in this pH range.

The amino acid residues D245, E273 and D343 (*B.* sp*.* G825-6 CGTase numbering) form the catalytic triad of the CGTase [1]. The residues E273 and D245 are involved in the initial cleavage of the α-1,4-linked gluco-oligosaccharide substrate [34,35]. In the first step of the CD synthesis reaction, E273 protonates the oxygen of the glycosidic bond of the substrate to form an intermediate [36]. The protonated state of E273 is formed by a hydrogen bond with D245 [37]. Upon substrate binding, the hydrogen bond is opened and E273 is deprotonated. In the second step of the reaction, the hydroxyl group of E273 is activated by deprotonation of an acceptor molecule. D343 performs a nucleophilic attack on the anomeric C1 atom of the donor molecule [38]. As previously shown for the CGTase from *Thermoanaerobacterium thermosulfurigensis* EM1 [27], mutations near these catalytic residues can be expected to affect the pH activity range of the enzyme. The variant S32 showed an increased CD_8_-synthesizing activity in the high pH range retaining more than 30% of its activity at pH 11.0. The substitution F158L of S32 is located close to the active site in a highly conserved region between the domains A1-B and in direct neighborhood to D245. The CGTases from *Bacillus agaradhaerens* and *Bacillus* sp. *BL31* CGTase also have a Leu residue at this position [8,10].

The variant S35 showed high activity in the low pH range up to pH 4.0. All of the nine amino acid substitutions of S35 were located distant from the active site. K39 is also found in several β-CGTases, e.g. *Bacillus* sp. G1 [39] and *Brevibacillus brevis* CD162 [40]. Both of these β-CGTases are stable in a range from pH 6.0 and 5.5 to pH 9.0 and 10.0, respectively. The substitutions T66S and L71P are located in random coil regions only about 13 Å away from the active site. Several CGTases have Ala, Asp, Glu, Gly, Asn, Pro, Gln and Ser in position 66. The residue L71 is conserved in almost all of them [14,27,31,39–57] with the exception of the CGTase from *Paenibacillus* sp. P22 [51] showing a Lys at this position. L71P is located 15 Å distant from the active site and in direct neighborhood to T72, a residue forming a part of the subsite −3 [31].

The residue I101 is conserved in all of the 30 CGTases examined for comparison [40]. In the variant S35, the substitution I101L is with a distance of only 11–14 Å located very closely to the active site. The substitutions S461G and E472G are located in the C-domain, while V605A, N606K and R648H are in the E-domain and thus much further away from the active site. However, these substitutions did obviously also contribute to the observed changes in pH activity of the CGTase (Fig. 2). The importance of these residues influencing the pH activity of a CGTase has not been reported previously and remains to be elucidated further. The effects of amino acid exchanges on the pH activity were observed both at low (two amino acid substitutions) and at medium mutagenic conditions (nine substitutions). A change of the pH activity range of an α-amylase by mutagenesis experiments has been reported previously [56]. From an error-prone random mutagenesis library containing 7200 clones, two variants showing this feature could be identified. The mutations were positioned both on conserved and non-conserved residues supporting our results that the pH activity range of the CGTase is determined by amino acids located at different locations in the enzyme. By site-directed mutagenesis at five positions based on sequence comparisons of cellulases with different pH optima, the pH activity of a cellobiohydrolase from the filamentous fungus *Trichoderma reesei* could be shifted to the alkaline pH range [58]. By using DNA shuffling and combinatorial mutations at different amino acid positions, the pH activity of a luciferase from *Photinus pyralis* and of a xylanase from *Themobifida fusca* [59] could also be successfully manipulated. These results further support the validity of our approach to employ DNA shuffling and error-prone PCR as suitable methods to change the pH activity range of CGTases or other enzymes without detailed structural information about the enzyme.

### 2.3 Product specificity of the CGTase variants

The ratio of CD_7_:CD_8_ synthesized by the γ-CGTase variants was determined. While the wild-type CGTase synthesized CD_7_ and CD_8_ in a ratio of 1:3 without the formation of CD_6_, several of the variants showed drastic changes in the ratios of the two CD products (Table 1). The substitution of Ser by Gly at position 184 in the variant S80 caused a shift in the CD_7_:CD_8_ ratio from 1:3 to 1:2. In comparison with the wild-type enzyme, the total yields obtained with these variants were however lower. The variants S42, S44 and S54 also showed a shift in the CD product ratio towards CD_8_ with about 80% CD_8_ and 20% CD_7_ produced concomitant with a 1.2 to 1.3-fold increase in CD_8_-synthesizing activity. The variant S77 showed with 91% the highest product specificity for CD_8_, however with a much reduced CD_8_-synthesizing activity of about 4% compared to the wild-type CGTase. Mutations affecting the product specificity for CD_8_ have previously been reported to result in decreased enzyme activities [1–3,59]. In this study, S42, S44 and S54 showed both an increase in product specificity and in CD_8_-synthesizing activity. These three variants share the substitutions N187D, A248V and V252E. The other 30 variants had Thr, Gln, Pro, Ser, Arg, Ala at position 187, but no Asp. A248V and V252E are located in a highly conserved loop region forming a part of the acceptor subsite +1 and +2. The catalytic residues D245 and E273 are also located in this region (Fig. 1B) [37]. The residue A248 has been predominantly found in γ-CGTases, whereas other CGTases have a Lys at this position. The mutations A248V and V252E are close to the catalytic site and are most likely involved in the detected increase in CD_8_-synthesizing activity and CD_8_ product specificity [40] of the three variants. The substitution with valine as another apolar amino acid at position 248 could be a significant modification contributing to the detected activity increase since it is located next to H249, a residue that plays an important role in cyclization activity [60]. The changes due to the introduction of V248 may have influenced the conformation of H249 in a way that the CD_8_-synthesizing activity increased. The more hydrophobic propyl group of Val may also have contributed to this effect due to an improved exclusion of water from the active site. This could result in an increase in CD_8_ yields since fewer water molecules could act as acceptors of the covalently bound substrate intermediates in a hydrolysis reaction of the CGTase. In addition, Val may have a positive influence on the conformation of the substrate intermediate by indirectly functioning as a steric barrier helping the non-reducing end of the substrate intermediate to bind at the acceptor subsite +1 position [37,61]. A site saturation mutagenesis at this position has been performed with the γ-CGTase from *Bacillus clarkii* 7364 [28]. The changes did not include substitutions with apolar amino acids like Val. The replacement of Ala by Arg or Lys at this position resulted in an increased product specificity for CD_8_ without reducing the synthesizing activity of the enzyme [28]. In contrast, the synthesizing activity of the CGTase from *Thermoanaerobacterium thermosulfurigenes* EM1 was decreased when a corresponding substitution has been introduced [62]. The substitution A246V (corresponding to the numbering in the G825-6 CGTase) in the CGTase from *Bacillus circulans* 251 resulted in decreased cyclization and increased hydrolysis activities of the enzyme [61]. The Ala in this position was found to be conserved in all of the compared CGTases [40].

The amino acid position 225 is not highly conserved in CGTases. The variants S63 S64, S69 and S77 showed a decreased CD_8_-synthesizing activity. They all carried the substitutions E145K, R225C and S461G. While E145 is typical for γ-CGTases [40], other CGTases also have Ala, Asp, Asn, Ser and Thr at this position. Instead of an Arg at position 225 Ala, Lys, Asn, Gln, Ser, Thr and Val but not Cys are found in different CGTases at this position. S461G is a substitution that also occurs in many CGTases including in the γ-CGTase of *B. clarkii* [40]. The mutation S461G is therefore unlikely to result in a negative effect on the CD_8_-synthesizing activity. E145K and R225C located at α-helices distant from the active site are therefore likely to be responsible for the detected decreased CD_8_-synthesizing activity of these variants.

Other mutations affecting the subsite −3 are G114D, F116L and D388E found in the variant S45 [63]. The subsite −3 is formed by amino acid residues located in four loops within the random coil region of the protein. One of these loops is formed by 112-HPGGFAS-118, a sequence typically found in γ-CGTases. D386 is located in a further loop and is also a part of the subsite −3. The mutation D388E may have affected D386 together with G114D and F116L resulted in a variant with a CD_8_-synthesizing activity reduced by 78%. The subsite −3 has been shown to contribute to the CD product specificity in CGTases [2,3]. The CD_7_:CD_8_ product ratio of S45 was indeed slightly shifted towards CD_7_ with a ratio of 1:1.

The substitution N194D in S42, located directly in front of the subsite −6, did not affect the CD_8_-synthesizing activity, but may have played a role in the detected increase of the product specificity for CD_8_. Other CGTases have Asn or Tyr in this position, while Phe was found in this position in the α-CGTase of *Anaerobranca gottschalkii* [40].

The residue Y174 is also conserved in CGTases [40]. While the substitution with His in S34 did not affect the CD_8_-synthesizing activity, its CD_7_:CD_8_ product ratio was shifted towards CD_7_ by 1:1. This variant also carried the substitution D151N. Many CGTases have a Gly at this position, whereas Asp is found in most γ-CGTases. In contrast, the α-CGTase of *B. macerans* [51] and the γCGTase of *Bacillus* sp. 1011 [48] have Asn in this position. Furthermore, D151 N is located far away from the subsite structures and therefore unlikely to be involved in influencing the product specificity of the enzyme for CD_8_.

## 3 Conclusions

CGTase variants were obtained by random mutagenesis with amino acid exchanges at subsites near and aloof of the catalytic site. The variants showed increased CD_8_ product specificity and a changed pH activity range. CGTases yielding CD_8_ as the main product and showing activity in the low pH range are useful biocatalysts for the industrial production of larger CD at competitive costs.

## 4 Materials and methods

### 4.1 Bacterial strains and plasmids

*Escherichia coli* BL21 (DE3) and pET-20b(+) were used for recombinant protein expression of the wild-type CGTase. *E. coli* One Shot Top 10 (Invitrogen) and pBADTOPO vector was used for production of mutant CGTase proteins.

### 4.2 Amplification and cloning of cgtS

The *cgtS* gene of the γ-CGTase from *Bacillus* sp. G-825-6 [14] was synthesized and codon-optimized for *E. coli* and *Bacillus subtilis* by Geneart (Regensburg, Germany). Standard polymerase chain reaction (PCR) was performed with *DreamTaq™* DNA polymerase (Fa. Thermo Scientific, Waltham, MA USA) using the cycle program: {pre-denaturation} 5 min at 95 °C, 29 × (45 s at 95 °C, 30 s at 61 °C and 90 s at 72 °C) and {final extension} 72 °C for 5 min. The *cgtS* gene was amplified with the primer pair fw-primer 5′-TTGATATCATGATTCGCCGCCTGAGC-3′ and rev-primer 5′-TTGAGCTCGACTGGTTATAATTCACTTCCACAATGC-3′ (Metabion, Martinsried, Germany). *Eco*RV and *Sac*I restriction sites (underlined) were added to the 5′ end of the primer, respectively. The stop codon of the gene was eliminated by the reverse primer and the PCR fragment was cloned into the expression vector pET20b(+) (Novagen, Darmstadt, Germany) using the restriction sites *Eco*RV and *Sac*I. The resulting open reading frame consisted of a 5′ pelB coding sequence, the *cgtS*-*Eco*RV/*Sac*I fragment and a 3′ His_6_-tag coding sequence. The construct was cloned into *E.coli* XL-1 blue cells. DNA sequencing confirmed the correct construction of the pET20b(+):*cgtS* and was performed by GATC Biotech (Konstanz, Germany). The pET20b(+):*cgtS* vector was then cloned into the expression strain *E. coli* BL21 (DE3).

### 4.3 Stepwise random mutagenesis

Two steps of mutagenesis were performed by error-prone PCR. Random mutagenesis was conducted according to the supplier’s manual using a Diversify PCR Random Mutagenesis Kit (Fa. Clontech, Mountain View, USA). Mutation rates of 2.7 mutations/kb and 3.5 mutations/kb were used. For a third step, a DNA shuffling procedure was performed. Template DNA from the second error-prone PCR with an identity of 98–99% was selected and digested with DNaseI (1 U/μl, RNase free, Thermo Scientific, Waltham, MA USA) into 200–500 bp fragments. Agarose gel-purified fragments served as template in a primer-less shuffling PCR with the following conditions: denaturation for 90 s at 95 °C, heterologous re-annealing (45×) for 30 s at 95 °C, 90 s at 65 °C, 90 s at 62 °C, 90 s at 59 °C, 90 s at 56 °C, 90 s at 53 °C, 90 s at 50 °C, 90 s at 47 °C, 90 s at 44 °C, 90 s at 41 °C, 90 s at 72 °C and terminal elongation for 420 s at 72 °C. The amplified product was directly used in a second PCR with the addition of specific forward and reverse primers. The complete open reading frame and flanking regions were sequenced by GATC Biotech (Konstanz, Germany).

### 4.4 Recombinant production and purification of the γ-CGTase and its variants

Batch cultivation of recombinant *E. coli* was performed in 3 l Luria–Bertani medium (LB medium, DSM 381) supplemented with 100 μg/ml ampicillin at 37 °C. Recombinant protein production was induced at an optical density of 1 (600 nm) by adding isopropyl β-d-1-thiogalactopyranoside (IPTG) to a final concentration of 1 mM. Cells were harvested by centrifugation (4 °C, 10.000*g*, 10 min), re-suspended in 100 mM sodium acetate/citrate/borate buffer (pH 8.5) and disrupted by sonication on ice in 3 cycles in 1 min, 120 W, 50% pulse and 50% power (Sonoplus ultrasonic homogenizer equipped with a UW 2200 ultrasonic head KE76, Bandelin, Berlin, Germany). The γ-CGTase in the soluble crude extract fraction was purified by affinity chromatography with 1 ml His Trap^™^ FF Crude Columns (GE Healthcare, Munich, Germany). Purification results were analyzed by sodium dodecyl sulfate–polyacrylamide gel electrophoresis [64] following the determination of protein concentration [65].

### 4.5 Determination of starch-hydrolyzing activity

The hydrolysis of starch by the enzymes was determined as described previously with 1% (w/v) soluble starch at pH 9.5 and 50 °C [66]. One unit of activity was defined as the amount of enzyme hydrolyzing 1 mg starch per 10 min.

### 4.6 *Determination of* CD_8_-synthesizing activity and CD_7_:CD_8_ ratios

Clones obtained from the random mutagenesis experiments were screened for the synthesis of CD_8_ on agar plates containing 10 g/l tryptone, 5 g/l yeast extract, 10 g/l soluble starch, 5 g/l NaCl, 0.1 g/l congored dye and 0.01 g/l xylencyanole (pH 7.0) [33]. Positive clones showed clear halos around the area of growth.

The enzymatically synthesized CD products were analyzed by high performance anion exchange chromatography with pulsed amperometric detection. Soluble starch (1%, 500 μl) in 50 mM phosphate buffer with a pH from 2.5 to 13.3 was incubated with purified γ-CGTase (40 μg/ml) for 4 h at 50 °C. The reaction was stopped by heating to 100 °C and the solution was adjusted to pH 6.0. Glucoamylase (250 mU/ml) (Sorachim, Lausanne, Switzerland) was added and the solution was incubated for 16 h at 60 °C to convert linear oligosaccharides and remaining starch to glucose. An ICS-3000 system (Dionex, Sunnyvale, USA) equipped with a CarboPac-PA100 column (4 × 250 mm) was used. The eluent buffer (A) contained 150 mM NaOH and the gradient buffer (B) 150 mM NaOH and 200 mM sodium nitrate. The following program was used: equilibration with 100% A for 10 min (1 ml/min), injection at −1.7 min, gradient I (0–12 min) 96% A/4% B, gradient II (12–30 min) 88% A/12% B, gradient III (30–31 min) 53% A/47% B and gradient IV (31–32 min) 100% B. The amounts of synthesized CD_7_ and CD_8_ were calculated using calibration curves (0.1–25 μg/ml CD) prepared with CD_7_ and CD_8_ standards (Wacker-Chemie GmbH, Burghausen, Germany). From the peak areas obtained, the concentrations of CD_7_ and CD_8_ were calculated based on three independent experiments. Each determination was performed in duplicate. The CD_8_-synthesizing activity of the wild-type γ-CGTase was 6.1 ± 0.45 nmol/min and its CD_7_:CD_8_ ratio was 1:3.

### 4.7 Amino acid sequence alignments and protein structure modeling

The amino acid sequence of the wild-type γ-CGTase G835–6 was compared with 30 other CGTase sequences. The sequences with the accession numbers WP_003323850.1, CAA01436.1, BAH14968.1, AAP31242.1, ABN14270.1, CAH61550.1, AEL33336.1, WP_022587620.1, AGT45478, P31746.1, P27036.2, O30565.1, AEO89319.1, AAV38118.2, ADY17981.1, BAA02380.1, AAV38117.1, AAD00555.1, P26827.2, P14014.1, AAC04359.1, AGR66230.1, P42279.1, *Paenibacillus* sp. T16 sequence, ETT36448.1, X66106.1, WP_021879762.1, WP_007544393.1, P05618.1, CAO05752.1, M19880.1 were obtained from the NCBI server. The alignment was performed using MEGA 5.1 [67] and the CLUSTALW algorithm using default settings (Table S1). Models of the γ-CGTase G825–6 and its variants were generated using SWISS-MODEL [68] with the protein database file 1cygA (X-ray structure of *Geobacillus stearothermophilus* CGTase at 2.5 Å resolution) as the template structure. For visualization of the structures, the PyMol molecular graphic system (v0.99, Schrödinger, LCC) was used. Superimposed substrate molecules were obtained from the protein database file 1cxkA (X-ray structure of *B. circulans* strain 251 CGTase at 2.5 Å resolution) [63].

## Author contribution statement

Contributed to project idea: CF, WZ

Planned experiments: SM, CS, WZ

Performed experiments: SM, CS

Analyzed data: SM, CS

Wrote the paper: SM, CS, WZ

## Figures and Tables

**Table 1:** Effects of amino acid substitutions obtained by random mutagenesis of the γ-CGTase G-825-6 on their CD_8_-synthesizing activity and CD_7_:CD_8_ product ratio. The CD_8_-synthesizing activity of the variants in relation to the wild-type enzyme (=1.0) are shown. The CD_8_-synthesizing activity of the wild-type enzyme was 6.1 ± 0.45 nmol/min and its CD_7_:CD_8_ ratio was 1:3.

Variant	Amino acid substitutions	CD_8_-synthesizing activity	CD_7_:CD_8_ ratio
S4	E145K, R225C, S461G, V605A, R684H	0.99	1:1
S12	Y174H, D384N	1.10	1:1
S31	N187D, Q219R, A248V, V252E, N394Y, T580A, I634T	0.16	1:1
S32	F158L, N454Y, E687G	0.38	1:4
S33	E60G, Q511R, N540D, N587D	1.01	1:1
S34	G114D, D151N, Y174H, N454Y, T610A, V641A	1.11	1:1
S35	E39K, T66S, L71P, I101L, S461G, E472G, V605A, N606K, R684H	1.13	1:2
S41	Y174H, D631G, Y662F	1.29	1:1
S42	N187D, N194D, M233L, A248V, V252E, D338E, N454D, N574D, Y664C, E687G	1.33	1:5
S44	N187D, A248V, V252E, N394Y, I634T	1.21	1:4
S45	G114D, F116L, N187D, N217S, D388E, N454D, S476G	0.22	1:1
S51	Y174H, N176D, D384N, D465G, M637K, E687G	0.22	1:2
S54	N187D, A248V, V252E, H352L, D465G, E560V, E687G	1.22	1:7
S55	N31I, I41V, I99T, S281A, L322P, L363P, K526R, D529G, K632R, E687G, T690S	0.18	1:1
S63	E145K, R225C, S461G, N544Y, V568A, N570S, F591L, D668G, N688D	0.15	1:4
S64	E145K, R225C, S461G, V554A, Q598L, S674C, E687G	0.29	1:1
S69	E145K, S461G, V605A, R684H	0.12	1:2
S77	E145K, R225C, F440L, S461G, V605A, R684H	0.04	1:10
S78	I405V, G422V, T579S	0.20	1:2
S80	S184G, Y662F, N670D	1.12	1:3
S84	G114D, F222I	0.83	1:2

## Appendix A Supplementary data

Supplementary Table S1 Multiple protein sequence alignment of the 31 CGTases. Nd, not determined.
