# Supplementary material for: Stepwise error-prone PCR and DNA shuffling changed the pH activity range and product specificity of the cyclodextrin glucanotransferase from an alkaliphilic Bacillus sp
Source: FEBS Open Bio. 2015 Jun 11;5:528–34. doi: 10.1016/j.fob.2015.06.002 (PMC4491590; doi:10.1016/j.fob.2015.06.002)
Supplement: Supplementary Table S1 — Multiple protein sequence alignment of the 31 CGTases. Nd, not determined. [file mmc1.docx]

**Supplementary material**

FEBSOPENBIO-D-15-00077

Stepwise error-prone PCR and DNA shuffling changed the pH activity range and product specificity of the cyclodextrin glucanotransferase from an alkaliphilic *Bacillus sp*.

**Table S1.** Multiple protein sequence alignment of the 31 CGTases. Nd, not determined.

| Acc.Nr. NCBI/PDB | Bacterial orign | Main CD product |
| --- | --- | --- |
| WP_003323850.1 | alkalophilic *Bacillus sp.* G-825-6 | γ |
| CAA01436.1 | *Bacillus firmus/lentus* 290-3 | β,γ |
| BAH14968.1 | *Bacillus clarkii* 7364 | γ |
| AAP31242.1 | *Bacillus agaradhaerens* LS-3C | β |
| ABN14270.1 | *Bacillus* sp. BL-31 | β |
| AAD00555.1 | *Geobacillus stearothermophilus* ET1 | β |
| P26827.2 | Thermoanaerobacterium  *thermosulfurigenes* Em1 | β |
| AEL33336.1 | *Bacillus* sp. 20R | β |
| WP_022587620.1 | *Caldanaerobacter suberraneus* | nd |
| CAH61550.1 | *Anaerobranca gottschalkii* | α |
| P27036.2 | *Bacillus obhensis* | β |
| AGT45478 | *Bacillus cereus* | nd |
| P31746.1 | *Bacillus* sp. 1-1 | β |
| AEO89319.1 | *Bacillus* sp. BPED101 | nd |
| O30565.1 | *Brevibacillus brevis* CD162 | β,γ |
| ADY17981.1 | *Bacillus sp*. NR5 UPM | nd |
| AAV38118.2 | *Bacillus sp.* G1 | β |
| BAA02380.1 | *Bacillus sp.* KC201 | β |
| AAC04359.1 | *Paenibacillus macerans* | α |
| AGR66230.1 | *Bacillus firmus* | β |
| P42279.1 | *Bacillus circulans* 251 | β |
| AAV38117.1 | *Bacillus sp.* TS1-1 | β |
| P05618.1 | alkalophilic *Bacillus sp*. 1011 | β |
|  | *Paenibacillus sp*. T16 | β,γ |
| ETT36448.1 | *Paenibacillus* sp. FSL R5-808 | nd |
| P31747.1 | *Bacillus* sp. 6.3.3 | nd |
| WP_021879762.1 | *Paenibacillus sp*. P22 | nd |
| WP_007544393.1 | *Haloferax larsenii* | nd |
| P14014.1 | *Bacillus licheniformis* | α.β |
| CAO05752.1 | *Paenibacillus pabuli* | nd |
| M19880.1 | *Bacillus sp. 38-2* | β |

**10 20 30 40 50**

**....|....|....|....|....|....|....|....|....|....|**

**B_sp_G825-6** 1 **-------------------MIRRLSFSLVVLFLISFLVIVN---------** 22

**_B_firmus_l** 1 **-------------------MIRRLSFSLVVLFLISFLVIVN---------** 22

**_B_clarkii_** 1 **-------------------MFRKLLCTLVTIITLSAWIVSHG-------G** 24

**_B_agaradha** 1 **--------------------------------------------------** 1

**B_sp_BL-31**  1 **---------------MRKKTLKRLLTLVVMLVILSGLSILDFSI-----T** 30

**G_stearothe** 1 **--------------------------------------------------** 1

**T_thermosul** 1 **--------------------------------------------------** 1

**B_sp_20RF**  1 **-------------------MKNLRFLMKTIP---LSLFLLLLL-------** 21

**C_subterran** 1 **-----------------MKKTLKLLSILLIT---IALLFSTIP-------** 23

**A_gottschal** 1 **------------MINKKNSIGKAICICLSILLLFGVLSIFQPVTNATQNS** 38

**B_obhensis**  1 **-------------------MKNLTVLLKTIP---LALLLFILL-------** 21

**B_cereus**  1 **----------------------------MIT---PSFIISSFL-------** 12

**B_sp_1-1**  1 **-------------------MNDLNDFLKTIL---LSFIFFLLL-------** 21

**B_sp_BPED10** 1 **-------------------MNCLNGFLKTIS---LCFIFFLLL-------** 21

**B_brevis_CD** 1 **----------------------------MIT---PSFIISSFL-------** 12

**B_sp_NR5_UP** 1 **-MFYFIYVLSQFVEGG-IELNCLNGFLKTIS---LCFIFFLLL-------** 38

**B_sp_G1-200** 1 **-------------------MNDLNDFLKTIS---LSFIFFLLL-------** 21

**B_sp_KC201**  1 **MADTWASEIGKLSQDKPFDLFSGKKVEKGVSGGITSFIFFLLL-------** 43

**P_macerans**  1 **-----------------MKSRYKRLTSLALS---LSMALGISL-------** 23

**B_firmus**  1 **-MFYFIYVLSQFVGGG-IELNDLNDLLKTIS---LSFIFFLLL-------** 38

**B_Circulans** 1 **--------------------------------------------------** 1

**B_sp_TS1-1**  1 **-MFCFIYVLSQFVEGG-IELNDLNDFLKTIS---LSFIFFLLL-------** 38

**B_sp_1011**  1 **--------------------------------------------------** 1

**P_sp_T16**  1 **-------------------MKRFMKLTAVWT---LWLSLTLGL-----LS** 23

**P_sp_FSL_R5** 1 **-------------------MKKFYKLTTALA---LSLSLTLSL-----FG** 23

**B_sp_6.3.3**  1 **----------------MFQMAKRAFLSTTLT---LGLLAGSAL-PFLPAS** 30

**P_sp_P22**  1 **---------------MLMNLTLKRILAGAAI---ATMLAGAQLTPAAPMG** 32

**H_larsenii**  1 **------------------MPFDRRTFLKGLGLSAIGIAGSTGAGGDDLAF** 32

**B_lichenifo** 1 **----------------MFQMAKRVLLSTTLT---FSLLAGSAL-PFLPAS** 30

**P_pabuli**  1 **----------------MFQMAKRVLLSTTLT---LSLLAGGAL-PFLPAS** 30

**B_sp_38-2**  1 **-------------------MKRFMKLTAVWT---LWLSLTLGL-----LS** 23

**60 70 80 90 100**

**....|....|....|....|....|....|....|....|....|....|**

**B_sp_G825-6** 22 **-PEYTEANENLDNVNYAEEIIYQIVTDRFYDGDPTNNPEGA-LFSTGCLD** 70

**_B_firmus_l** 22 **-PEYTEANENLDNVNYAQEIIYQIVTDRFYDGDPTNNPEGT-LFSPGCLD** 70

**_B_clarkii_** 25 **EVHASNATNDLSNVNYAEEVIYHIVTDRFKDGDPDNNPQGQ-LFSNGCSD** 73

**_B_agaradha** 1 **----QQATDRSNSVNYSTDVIYQIVTDRFYDGDESNNPSGE-LYSEDCKN** 45

**B_sp_BL-31**  31 **SASAQQATDRSNSVNYSTDVIYQIVTDRFYDGDESNNPSGE-LYSEDCKN** 79

**G_stearothe** 1 **-------AGNLNKVNFTSDVVYQIVVDRFVDGNTSNNPSGA-LFSSGCTN** 42

**T_thermosul** 1 **----ASDTAVSNVVNYSTDVIYQIVTDRFVDGNTSNNPTGD-LYDPTHTS** 45

**B_sp_20RF**  22 **SLTTVAEADVTNKVDYSKDVIYQIVTDRFSDGDPSNNPTGA-IYSQGCSD** 70

**C_subterran** 24 **SVPAAPDTSVSNVVNYSTDVIYQIVTDRFLDGNPNNNPTGD-LYDPTHTS** 72

**A_gottschal** 39 **LEHIKEHTSVNNQVNYATDVIYQIVTDRFLDGDKYNNPTCENLYSEDGAD** 88

**B_obhensis**  22 **SLPTAAQADVTNKVNYTRDVIYQIVTDRFSDGDPSNNPTGA-IYSQDCSD** 70

**B_cereus**  13 **SLPTVVEASVTNKVNYSKDVIYQIVTDRFSDGNPANNPSGA-IFSQNCSD** 61

**B_sp_1-1**  22 **SLPTVAEADVTNKVNYSKDVIYQIVTDRFSDGNPGNNPSGA-IFSQNCID** 70

**B_sp_BPED10** 22 **SLPTVAEADVTNKVNYSKDVIYQIVTDRFSDGNPGNNPSGA-IYSQNCID** 70

**B_brevis_CD** 13 **SLPTVVEASVTNKVNYSKDVIYQIVTDRFSDGNPANNPSGA-IFSQNCSD** 61

**B_sp_NR5_UP** 39 **SLPTVAEADVTNKVNYSKDVIYQIVTDRFSDGNPGNNPSGA-IYSQNCID** 87

**B_sp_G1-200** 22 **SLPTVAEADVTNKVNYSKDVIYQVVTDRFSDGNPGNNPSGA-IFSQNCID** 70

**B_sp_KC201**  44 **SLPTVAEADVTNKVNYSKDVIYQIVTDRFSDGNPGNNPSGA-IFSQNCID** 92

**P_macerans**  24 **PAWASPDTSVDNKVNFSTDVIYQIVTDRFADGDRTNNPAGD-AFSGDRSN** 72

**B_firmus**  39 **SLPTVAEADVTNKVNYSKDVIYQVVTDRFSDGNPGNNPSGA-IFSQNCID** 87

**B_Circulans** 1 **----APDTSVSNKQNFSTDVIYQIFTDRFSDGNPANNPTGA-AFDGTCTN** 45

**B_sp_TS1-1**  39 **SLPTVAEADVTNKVNYSKDVIYQVVTDRFSDGNPGNNPSGA-IFSQNCID** 87

**B_sp_1011**  1 **----APDTSVSNKQNFSTDVIYQIFTDRFSDGNPANNPTGA-AFDGSCTN** 45

**P_sp_T16**  24 **PVHAAPDTSVSNKQNFSTDVIYQIFTDRFSDGNPANNPTGA-AFDGSCTN** 72

**P_sp_FSL_R5** 24 **PAYAAPDTSVSNKQNFSTDVIYQIFTDRFSDGNASNNPTGS-AFDGTCTN** 72

**B_sp_6.3.3**  31 **AAYADPDIAVTNKQSFSTDVIYQVFTDRFLDGNPSNNPTGA-AYDATCSN** 79

**P_sp_P22**  33 **LASAAPDTAVGNKQNYSTDVIYQLFTDRFSDGNAANNPTGA-AFSSGCTN** 81

**H_larsenii**  33 **VSQASAASPPGHAVNFADDVIYQIISDRFHDGNSGNNPTGE-LGSDSCSN** 81

**B_lichenifo** 31 **AIYADADTAVTNKQNFSTDVIYQVFTDRFLDGNPSNNPTGA-AFDGTCSN** 79

**P_pabuli**  31 **AIYADADTAVTNKQNFSTDVIYQIFTDRFLDGNPSNNPTGA-AYDATCSN** 79

**B_sp_38-2**  24 **PVHAAPDTSVSNKQNFSTDVIYQIFTDRFSDGNPANNPTGA-AFDGSCTN** 72

**110 120 130 140 150**

**....|....|....|....|....|....|....|....|....|....|**

**B_sp_G825-6** 71 **LTKYCGGDWQGIIEKIEDGYLPDMGITAIWISPPIENVMELHPGGF----** 116

**_B_firmus_l** 71 **LTKYCGGDWQGVIEKIEDGYLPDMGITAIWISPPIENVMELHPGGF----** 116

**_B_clarkii_** 74 **LTKYCGGDWQGIIDEIESGYLPDMGITALWISPPVENVFDLHPEGF----** 119

**_B_agaradha** 46 **LRKYCGGDWQGIIDKIDDGYLTNMGVTALWISPPVENIFETIDDEFGT--** 93

**B_sp_BL-31**  80 **LRKYCGGDWQGIIDKIDDGYLTNMGVTALWISPPVENIFETIDDEFGT--** 127

**G_stearothe** 43 **LRKYCGGDWQGIINKINDGYLTDMGVTAIWISQPVENVFSVMNDASGS--** 90

**T_thermosul** 46 **LKKYFGGDWQGIINKINDGYLTGMGVTAIWISQPVENIYAVLPDSTFGGS** 95

**B_sp_20RF**  71 **LHKYCGGDWQGIIDKINDGYLTDLGITAIWISQPVENVYALHP-SGYT--** 117

**C_subterran** 73 **LKKYFGGDWQGIINKINDGYLTGMGITAIWISQPVENIYAVLPDSTFGGS** 122

**A_gottschal** 89 **LRKYLGGDWRGIIQKIEDGYLPDMGISAIWISSPVENIYAVHPQFG----** 134

**B_obhensis**  71 **LHKYCGGDWQGIIDKINDGYLTDLGITAIWISQPVENVYALHP-SGYT--** 117

**B_cereus**  62 **LHKYCGGDWQGIINKMNDGYLTDLGITALWISQPVENVYALHP-SGYT--** 108

**B_sp_1-1**  71 **LHKYCGGDWQGIIDKINDGYLTDLGITALWISQPVENVYALHP-SGYT--** 117

**B_sp_BPED10** 71 **LHKYCGGDWQGIIDKINDGYLTDLGITALWISQPVENVYALHP-SGYT--** 117

**B_brevis_CD** 62 **LHKYCGGDWQGIINKMNDGYLTDLGITALWISQPVENVYALHP-SGYT--** 108

**B_sp_NR5_UP** 88 **LHKYCGGDWQGIIDKINDGYLTDLGITALWISQPVENVYALHP-SGYT--** 134

**B_sp_G1-200** 71 **LHKYCGGDWQGIIDKINDGYLTDLGITALWISQPVENVYALHP-SGYT--** 117

**B_sp_KC201**  93 **LHKYCGGDWQGIIDKINDGYLTDLGITALWISQPVENVYALHP-SGYT--** 139

**P_macerans**  73 **LKLYFGGDWQGIIDKINDGYLTGMGVTALWISQPVENITSVIKYSGVN-N** 121

**B_firmus**  88 **LHKYCGGDWQGIIDKINDGYLTDLGITALWISQPVENVYALHP-SGYT--** 134

**B_Circulans** 46 **LRLYCGGDWQGIINKINDGYLTGMGVTAIWISQPVENIYSIINYSGVN-N** 94

**B_sp_TS1-1**  88 **LHKYCGGGWQGIIDKINDGYLTDLGITALWISQPVENVYALHP-SGYT--** 134

**B_sp_1011**  46 **LRLYCGGDWQGIINKINDGYLTGMGITAIWISQPVENIYSVINYSGVN-N** 94

**P_sp_T16**  73 **LRLYCGGDWQGIINKINDGYLTGMGITAIWISQPVENIYSVINYSGVH-N** 121

**P_sp_FSL_R5** 73 **LRLYCGGDWQGIINKINDGYLTGMGVTAIWISQPVENIYSVINYSGVN-N** 121

**B_sp_6.3.3**  80 **LKLYCGGDWQGLINKINDNYFSDLGVTALWISQPVENIFATINYSGVT-N** 128

**P_sp_P22**  82 **KRVYCGGDWQGIVNKINDNYFTGMGVTALWISQPVENIYSVINYSGVT-N** 130

**H_larsenii**  82 **LRKYCGGDWQGIIDKIQSGYLTDLGVSAIWISPPFENITAVDSDIGTS--** 129

**B_lichenifo** 80 **LKLYCGGDWQGLVNKINDNYFSDLGVTALWISQPVENIFATINYSGVT-N** 128

**P_pabuli**  80 **LKLYCGGDWQGLINKINDNYFSDLGVTALWISQPVENIFATINYGGVI-N** 128

**B_sp_38-2**  73 **LRLYCGGDWQGIINKINDGYLTGMGITAIWISQPVENIYSVINYSGVH-N** 121

**160 170 180 190 200**

**....|....|....|....|....|....|....|....|....|....|**

**B_sp_G825-6** 117 **ASYHGYWGRDFKRTNPAFGSLADFSRLIETAHNYDIKVIIDFVPNHTSPV** 166

**_B_firmus_l** 117 **ASYHGYWGRDFKRTNPAFGSLADFSRLIETAHNHDIKVIIDFVPNHTSPV** 166

**_B_clarkii_** 120 **SSYHGYWARDFKKTNPFFGDFDDFSRLIETAHAHDIKVVIDFVPNHTSPV** 169

**_B_agaradha** 94 **TSYHGYWARDYKKTNPFFGSTEDFERLIETAHSHDIKIVIDLAPNHTSPA** 143

**B_sp_BL-31**  128 **TSYHGYWARDYKKTNPFFGSTEDFERLIETAHSHDIKIVIDLAPNHTSPA** 177

**G_stearothe** 91 **ASYHGYWARDFKKPNPFFGTLSDFQRLVDAAHAKGIKVIIDFAPNHTSPA** 140

**T_thermosul** 96 **TSYHGYWARDFKRTNPYFGSFTDFQNLINTAHAHNIKVIIDFAPNHTSPA** 145

**B_sp_20RF**  117 **-SYHGYWARDYKRTNPFYGDFSDFDRLIDTAHGNGIKVIMDFTPNHSSTA** 166

**C_subterran** 123 **TSYHGYWARDFKKTNPFFGSFTDFKNLIATAHAHNIKVIIDFAPNHTSPA** 172

**A_gottschal** 135 **TSYHGYWARDFKRNNPFFGDLNDFRELIAVANEHDIKVIIDFAPNHTSPA** 184

**B_obhensis**  117 **-SYHGYWARDYKRTNPFYGDFSDFDRLMDTAHSNGIKVIMDFTPNHSSPA** 166

**B_cereus**  108 **-SYHGYWARDYKKTNPYFGNFSDFDRLVSTAHNKGIKIIMDFTPNHSSPA** 157

**B_sp_1-1**  117 **-SYHGYWARDYKKTNPYYGNFDDFDRLMSTAHSNGIKVIMDFTPNHSSPA** 166

**B_sp_BPED10** 117 **-SYHGYWARDYKKTNPYYGNFDDFDRLMSTAHNNGIKVIMDFTPNHSSPA** 166

**B_brevis_CD** 108 **-SYHGYWARDYKKTNPYFGNFSDFDRLVSTAHNKGIKIIMDFTPNHSSLA** 157

**B_sp_NR5_UP** 134 **-SYHGYWARDYKKTNPYYGNFDDFDRLMSTAHNNGIKVIMDFTPNHSSPA** 183

**B_sp_G1-200** 117 **-SYHGYWARDYKKTNPYYGNFDDFDRLMSTAHSNGIKVIMDFTPNHSSPA** 166

**B_sp_KC201**  139 **-SYHGYWARDYKKTNPYYGNFDDFDRLMSTAHSNGIKVIMDFTPNHSSPA** 188

**P_macerans**  122 **TSYHGYWARDFKQTNDAFGDFADFQNLIDTAHAHNIKVVIDFAPNHTSPA** 171

**B_firmus**  134 **-SYHGYWARDYKKTNPYYGNFDDFDRLMSTAHSNGIKVIMDFTPNHSSPA** 183

**B_Circulans** 95 **TAYHGYWARDFKKTNPAYGTIADFQNLIAAAHAKNIKVIIDFAPNHTSPA** 144

**B_sp_TS1-1**  134 **-SYHGYWARDYKKTNPYYGNFDDFDRLMSTAHSNGIKVIMDFTPNHSSPA** 183

**B_sp_1011**  95 **TAYHGYWARDFKKTNPAYGTMQDFKNLIDTAHAHNIKVIIDFAPNHTSPA** 144

**P_sp_T16**  122 **TAYHGYWARDFKKTNPAYGTMQDFKNLIDTAHAHNIKVIIDFAPNHTSPA** 171

**P_sp_FSL_R5** 122 **TAYHGYWARDFKKTNPAYGTIADFQNLITAAHAKNIKVIIDFAPNHTSPA** 171

**B_sp_6.3.3**  129 **TAYHGYWARDFKKTNPYFGTMADFQNLITTAHAKGIKIIIDFAPNHTSPA** 178

**P_sp_P22**  131 **TAYHGYWARDFKKTNPAFGSFTDFQNLINAAHAKGIKVIIDFAPNHTSPA** 180

**H_larsenii**  129 **--YHGYWARDFTDANQFFGDMETFEQLVSVAHQNDIKVVIDFVPNHTSPS** 177

**B_lichenifo** 129 **TAYHGYWARDFKKTNPYFGTMTDFQNLVTTAHAKGIKIIIDFAPNHTSPA** 178

**P_pabuli**  129 **TAYHGYWARDFKKTNPYFGTMADFQNLITTAHAKGIKIVIDFAPNHTSPA** 178

**B_sp_38-2**  122 **TAYHGYWARDFKKTNPAYGTMQDFKNLIDTAHAHNIKVIIDFAPNHTSPA** 171

**210 220 230 240 250**

**....|....|....|....|....|....|....|....|....|....|**

**B_sp_G825-6** 167 **D------IEDGALYDNGRLVGHYSNDNEDYFYTNGGSDFSSYEDSIYRNL** 210

**_B_firmus_l** 167 **D------IENGALYDNGRLVGHYSNDSEDYFYTNGGSDFSSYEDSIYRNL** 210

**_B_clarkii_** 170 **D------IEDGALYDNGTLLGHYSTDANNYFYNYGGSDFSDYENSIYRNL** 213

**_B_agaradha** 144 **DFDNPDYAENGVLYDDGNYLGSYSDDSDLFLYN-GGTDFSNYEDEIYRNL** 192

**B_sp_BL-31**  178 **DFDNPDYAENGVLYDDGNYVGSYSDDSDLFLYN-GGTDFSNYEDEIYRNL** 226

**G_stearothe** 141 **SETNPSYMENGRLYDNGTLLGGYTNDANMYFHHNGGTTFSSLEDGIYRNL** 190

**T_thermosul** 146 **SETDPTYAENGRLYDNGTLLGGYTNDTNGYFHHYGGTDFSSYEDGIYRNL** 195

**B_sp_20RF**  167 **LETDPTYAENGAIYDDGVLLGNYSNDPLNLFHHNGGTDFSSYEDSIYRNL** 216

**C_subterran** 173 **SETDPTYGENGRLYDNGVLLGGYTNDTNGYFHHYGGTNFSSYEDGIYRNL** 222

**A_gottschal** 185 **EVNNPNYAEDGNLYNNGEFVASYSNDLNEIFYHFGGTDFSTYEDSIYRNL** 234

**B_obhensis**  167 **LETDPSYAENGAVYNDGVLIGNYSNDPNNLFHHNGGTDFSSYEDSIYRNL** 216

**B_cereus**  158 **LETNPNYVENGALYNNGALLGNYSNDRNKLFHHNGGTDFSSYEDSIYRNL** 207

**B_sp_1-1**  167 **LETNPNYVENGAIYDNGALLGNYSNDQQNLFHHNGGTDFSSYEDSIYRNL** 216

**B_sp_BPED10** 167 **LETNPNYVENGAIYDNGALLGNYSNDQQNLFHHNGGTDFSSYEDSIYRNL** 216

**B_brevis_CD** 158 **LETNPNYVENGALYNNGALLGNYSNDRNKLFHHNGGTDFSSYEDSIYRNL** 207

**B_sp_NR5_UP** 184 **LETNPNYVENGAIYDNGALLGNYSNDQQNLFHHNGGTDFSSYEDSIYRNL** 233

**B_sp_G1-200** 167 **LETNPNYVENGAIYDNGTLLGNYSNDQQNLFHHNGGTDFSSYEDSIYRNL** 216

**B_sp_KC201**  189 **LETNPNYVENGAIYDNGALLGNYSNDQQNLFHHNGGTDFSSYEDSIYRNL** 238

**P_macerans**  172 **DRDNPGFAENGALYDNGSLLGAYSNDTAGLFHHNGGTDFSTIEDGIYKNL** 221

**B_firmus**  184 **LETNPNYVENGAIYDNGALLGNYSNDQQNLFHHNGGTDFSSYEDSIYRNL** 233

**B_Circulans** 145 **SSDQPSFAENGRLYDNGTLLGGYTNDTQNLFHHNGGTDFSTTENGIYKNL** 194

**B_sp_TS1-1**  184 **LETNPNYVENGAIYDNGTLLGNYSNDQQNLFHHNGGTDFSSYEDSIYRNL** 233

**B_sp_1011**  145 **SSDDPSFAENGRLYDNGNLLGGYTNDTQNLFHHYGGTDFSTIENGIYKNL** 194

**P_sp_T16**  172 **SSDDPSFAENGRLYDNGNLLGGYTNDTQNLFHHYGGTDFSTIENGIYKNL** 221

**P_sp_FSL_R5** 172 **SSDQPSFAENGKLYNNGTLLGGYTGDTQNLFHHNGGTDFSTTENGIYKNL** 221

**B_sp_6.3.3**  179 **METDTSFAENGKLYDNGTLVGGYTNDTNGYFHHNGGSDFSSLENGIYKNL** 228

**P_sp_P22**  181 **METDTSFAENGKLYNNGTLLGGYTNDTNGLFHHNGGSDFSTLENGIYKNL** 230

**H_larsenii**  178 **TSDDE--LEDGVLYDNGSYVAAYNDDPESYFHHNGGTDYSSYEGQIYRNL** 225

**B_lichenifo** 179 **METDTSFAENGKLYDNGNLVGGYTNDTNGYFHHNGGSDFSTLENGIYKNL** 228

**P_pabuli**  179 **METDTSFAENGKLYDNGNLVGGYTNDTNGYFHHNGGSDFSSLENGIYKNL** 228

**B_sp_38-2**  172 **SSDDPSFAENGRLYDNGNLLGGYTNDTQNLFHHYGGTDFSTIENGIYKNL** 221

**260 270 280 290 300**

**....|....|....|....|....|....|....|....|....|....|**

**B_sp_G825-6** 211 **YDLASLNQQNSFIDRYLKEAIQMWLDLGIDGIRVDAVAHMPVGWQKNFVS** 260

**_B_firmus_l** 211 **YDLASLNQQNSFIDRYLKESIQMWLDLGIDGIRVDAVAHMPVGWQKNFVS** 260

**_B_clarkii_** 214 **YDLASLNQQHSFIDKYLKESIQLWLDTGIDGIRVDAVAHMPLGWQKAFIS** 263

**_B_agaradha** 193 **FDLASFNHINSELNNYLEDAVKKWLDLGIDGIRIDAVAHMPPGWQKAYMD** 242

**B_sp_BL-31**  227 **FDLASFNHINPELNNYLEDAVKKWLDLGIDGIRVDAVAHMPPGWQKAYMD** 276

**G_stearothe** 191 **FDLADLNHQNPVIDRYLKDAVKMWIDMGIDGIRMDAVKHMPFGWQKSLMD** 240

**T_thermosul** 196 **FDLADLNQQNSTIDSYLKSAIKVWLDMGIDGIRLDAVKHMPFGWQKNFMD** 245

**B_sp_20RF**  217 **YDLADYNLNNDIIDQYLKESIKLWLDKGIDGIRVDAVKHMSEGWQTALMS** 266

**C_subterran** 223 **FDLADLDQQNNTIDSYLKAAIKLWLDMGIDGIRMDAVKHMAFGWQKNFMD** 272

**A_gottschal** 235 **FDLAGLNLNNNFVDQYLRDSIKFWLDLGVDGIRVDAVKHMPLGWQKSFVD** 284

**B_obhensis**  217 **YDLADYDLNNTVMDQYLKESIKLWLDKGIDGIRVDAVKHMSEGWQTSLMS** 266

**B_cereus**  208 **YDLADYDLNNKVVDQYLKESIKLWLDKGIDGIRVDAVKHMSEGWQTSLMS** 257

**B_sp_1-1**  217 **YDLADYDLNNTVMDQYLKESIKFWLDKGIDGIRVDAVKHMSEGWQTSLMS** 266

**B_sp_BPED10** 217 **YDLADYDLNNTVMDQYLKESIKFWLDKGIDGIRVDAVKHMSEGWQTSLMS** 266

**B_brevis_CD** 208 **YDLADYDLNNKVVDQYLKESIKLWLIK-IDGIRVDAVKHMSEGWQTSLMS** 256

**B_sp_NR5_UP** 234 **YDLADYDLNNTVMDQYLKESIKFWLDKGIDGIRVDAVKHMSEGWQTSLMS** 283

**B_sp_G1-200** 217 **YDLADYDLNNTVMDQYLKESIKFWLDKGIDGIRVDAVKHMSEGWQTSLMS** 266

**B_sp_KC201**  239 **YDLADYDLNNTVMDQYLKESIKFWLDKGIDGIRVDAVKHMSEGWQTSLMS** 288

**P_macerans**  222 **YDLADINHNNNAMDAYFKSAIDLWLGMGVDGIRFDAVKHMPFGWQKSFVS** 271

**B_firmus**  234 **YDLADYDLNNTAMDQYLKESIKFWLDKGIDGIRVDAVKHMSEGWQTSLMS** 283

**B_Circulans** 195 **YDLADLNHNNSTVDVYLKDAIKMWLDLGIDGIRMDAVKHMPFGWQKSFMA** 244

**B_sp_TS1-1**  234 **YDLADYDLNNTVMDQYLKESIKFWLDKGIDGIRVDAVKHMSEGWQTSLMS** 283

**B_sp_1011**  195 **YDLADLNHNNSSVDVYLKDAIKMWLDLGVDGIRVDAVKNMPFGWQKSFMA** 244

**P_sp_T16**  222 **YDLADLNHNNSSVDVYLKDAIKMWLDLGVDGIRVDAVKHMPFGWQKSFMS** 271

**P_sp_FSL_R5** 222 **YDLADLNHNNSTVDTYLKDAVKMWLDLGIDGIRMDAVKHMPFGWQKSFMA** 271

**B_sp_6.3.3**  229 **YDLADFNHNNATIDKYFKDAIKLWLDMGVDGIRVDAVKHIALGWQKSWMS** 278

**P_sp_P22**  231 **YDLADLEQNNSTIDTYFKDAIKVWLDLGIDGIRVDAAKHMSMGWQKNWVA** 280

**H_larsenii**  226 **YNLADFDQHETYIDQYLKDAIEQWLDTGIDGIRVDAVAHMPPKWQKTLVD** 275

**B_lichenifo** 229 **YDLADLNHNNSTIDTYFKDAIKLWLDMGVDGIRVDAVKHMPQGWQKNWMS** 278

**P_pabuli**  229 **YDLADLNHNNSTIDQYFKDAIKLWLDMGVDGIRVDAVKHMPLGWQKSWMS** 278

**B_sp_38-2**  222 **YDLADLNHNNSSVDVYLKDAIKMWLDLGVDGIRVDAVKHMPFGWQKSFMS** 271

**310 320 330 340 350**

**....|....|....|....|....|....|....|....|....|....|**

**B_sp_G825-6** 261 **SIY-DYNPVFTFGEWFTGASGSD-EYH-YFINNSGMSALDFRYAQVVQDV** 307

**_B_firmus_l** 261 **SIY-DYNPVFTFGEWFTGAGGSD-EYH-YFINNSGMSALDFRYAQVVQDV** 307

**_B_clarkii_** 264 **SVY-DYNPVFTFGEWFTGAQGSN-HYH-HFVNNSGMSALDFRYAQVAQDV** 310

**_B_agaradha** 243 **TIY-DHRAVFTFGEWFTGPSGNE-DYT-KFANNSGMSVLDFRFAQTTRNV** 289

**B_sp_BL-31**  277 **TIY-DHRAVFTFGEWFTGPNGNE-DYT-RFANNSGMSVLDFRFAQTTRNV** 323

**G_stearothe** 241 **EID-NYRPVFTFGEWFLSENEVDANNH-YFANESGMSLLDFRFGQKLRQV** 288

**T_thermosul** 246 **SIL-SYRPVFTFGEWFLGTNEIDVNNT-YFANESGMSLLDFRFSQKVRQV** 293

**B_sp_20RF**  267 **DIY-GYKPVFTFGEWFLGAGEVDPQNH-HFANESGMSLLDFQFGQTIREV** 314

**C_subterran** 273 **SIL-SYRPVFTFGEWYLGANEVDPNNT-YFANESGMSLLDFRFAQKVRQV** 320

**A_gottschal** 285 **TIY-NHKPVFVFGEWYLGKDEYDPNYY-HFANNSGMSLLDFEFAQTTRSV** 332

**B_obhensis**  267 **DIY-AHEPVFTFGEWFLGSGEVDPQNH-HFANESGMSLLDFQFGQTIRDV** 314

**B_cereus**  258 **DIY-TYKPVFTFGEWFLGTGEVDPQNH-HFANESGMSLLDFQFGQTIRSV** 305

**B_sp_1-1**  267 **EIY-SHKPVFTFGEWFLGSGEVDPQNH-HFANESGMSLLDFQFGQTIRNV** 314

**B_sp_BPED10** 267 **EIY-SHKPVFTFGEWFLGSGEVDPQNH-HFANESGMSLLDFQFGQTIRNV** 314

**B_brevis_CD** 257 **DIY-TYKPVFTFGEWFLGTGEVDPQNH-HFANESGMSLLDFQFGQTIRSV** 304

**B_sp_NR5_UP** 284 **EIY-SHKPVFTFGEWFLGSVEVDPQNH-HFANESGMSLLDFQFGQTIRNV** 331

**B_sp_G1-200** 267 **EIY-SHKPVFTFGEWFLGSGEVDPQNH-HFANESGMSLLDFQFGQTIRNV** 314

**B_sp_KC201**  289 **EIY-SHKPVFTFGEWFLGSGEVDPQNH-HFANESGMSLLDFQFGQTIRNV** 336

**P_macerans**  272 **SIYGGDHPVFTFGEWYLGADQTDGDNI-KFANESGMNLLDFEYAQEVREV** 320

**B_firmus**  284 **EIY-SHKPVFTFGEWFLGSGEVDPQNH-HFANESGMSLLDFQFGQTIRNV** 331

**B_Circulans** 245 **AVN-NYKPVFTFGEWFLGVNEVSPENH-KFANESGMSLLDFRFAQKVRQV** 292

**B_sp_TS1-1**  284 **EIY-SHKPVFTFGEWFLGSGEVDPQNH-HFANESGMSLLDFQFGQTIRNV** 331

**B_sp_1011**  245 **TIN-NYKPVFTFGEWFLGVNEISPEYH-QFANESGMSLLDFRFAQKARQV** 292

**P_sp_T16**  272 **TIN-NYKPVFTFGEWFLGVNEISPEYHNQFANESGMSLLDFRFAQKARQV** 320

**P_sp_FSL_R5** 272 **TVN-NYKPVFTFGEWFLGVNEVSAENH-KFANESGMSLLDFRFAQKVRQV** 319

**B_sp_6.3.3**  279 **SIY-VHKPVFTFGEWFLGSAASDADNT-DFANKSGMSLLDFRFNSAVRNV** 326

**P_sp_P22**  281 **SIY-GYKPVFVFGEWFMGSAAAEADNT-KFANESGMSLLDFRFNQEVRSV** 328

**H_larsenii**  276 **TIY-DHKPVFTFGEWFLGADQSNPRYY-EFSNDSGMSLLDFRFGQEIRQV** 323

**B_lichenifo** 279 **SIY-AHKPVFTFGEWFLGSAAPDADNT-DFANESGMSLLDFRFNSAVRNV** 326

**P_pabuli**  279 **SIY-AHKPVFTFGEWFLGSAASDADNT-EFANESGMSLLDFRFNSAVRNV** 326

**B_sp_38-2**  272 **TIN-NYKPVFNFGEWFLGVNEISPEYH-QFANESGMSLLDFPFAQKARQV** 319

**360 370 380 390 400**

**....|....|....|....|....|....|....|....|....|....|**

**B_sp_G825-6** 308 **LRNNDGTMYDLETVLRETESVYDKPQDQVTFIDNHDIDRFSRSGHSTRST** 357

**_B_firmus_l** 308 **LRNNDGTMYDLETVLRETESVYEKPQDQVTFIDNHDINRFSRNGHSTRTT** 357

**_B_clarkii_** 311 **LRNQKGTMHDIYDMLASTQLDYERPQDQVTFIDNHDIDRFTVEGRDTRTT** 360

**_B_agaradha** 290 **IGNNNGTMYDIEKMLTDTENDYDRPQDQVTFLDNHDMSRFTNGGESTRTT** 339

**B_sp_BL-31**  324 **IGNNNGTMYDIEAMLTDTENDYDRPQDQVTFLDNHDMSRFTNNGESTRTT** 373

**G_stearothe** 289 **LRNNSDNWYGFNQMIQDTASAYDEVLDQVTFIDNHDMDRFMIDGGDPRKV** 338

**T_thermosul** 294 **FRDNTDTMYGLDSMIQSTASDYNFINDMVTFIDNHDMDRFYNGG-STRPV** 342

**B_sp_20RF**  315 **LKDGNSDWYAFNEMIESTEEDYDEVIDQVTFIDNHDMSRFSIENSSNRHT** 364

**C_subterran** 321 **FRDNTDTMYGLDSMIQSTAADYNFINDMVTFIDNHDMDRFYTGG-STRPV** 369

**A_gottschal** 333 **FRNHEKNMFDLYDMLKNTENNYERVVDQVTFIDNHDMDRFHYDGATKRNV** 382

**B_obhensis**  315 **LMDGSSNWYDFNEMIASTEEDYDEVIDQVTFIDNHDMSRFSFEQSSNRHT** 364

**B_cereus**  306 **LKDRTSNWYDFNEMIKSTEKDYDEVIDQVTFIDNHDMSRFSMGSSSNRQT** 355

**B_sp_1-1**  315 **LKDRTSNWYDFNEMITSTEKEYNEVIDQVTFIDNHDMSRFSVGSSSNRQT** 364

**B_sp_BPED10** 315 **LKDRTSNWYDFHDMIKSTEKEYNEVIDQVTFIDNHDMSRFSVGSSSNRQT** 364

**B_brevis_CD** 305 **LKDRTSNWYDFNEMIKSTEKDYDEVIDQVTFIDNHDMSRFSM-VVFNFQT** 353

**B_sp_NR5_UP** 332 **LKDRTSNWYDFHDMIKSTEKEYNEVIDQVTFIDNHDMSRFSVGSSSNRQT** 381

**B_sp_G1-200** 315 **LKDRTSNWYDFNEMITSTEKEYNEVIDQVTFIDNHDMSRFSVGSSSNRQT** 364

**B_sp_KC201**  337 **LKDRTSNWYDFNEMITSTEKEYNEVIDQVTFIDNHDMSRFSVGSSSNRQT** 386

**P_macerans**  321 **FRDKTETMKDLYEVLASTESQYDYINNMVTFIDNHDMDRFQVAGSGTRAT** 370

**B_firmus**  332 **LKDRTSNWYDFNEMITSTEKEYNEVIDQVTFIDNHDMSRFSVGSSSNRQT** 381

**B_Circulans** 293 **FRDNTDNMYGLKAMLEGSAADYAQVDDQVTFIDNHDMERFHASNANRRKL** 342

**B_sp_TS1-1**  332 **LKDRTSNWYDFNEMITSTEKEYNEVIDQVTFIDNHDMSRFSVGSSSNRQT** 381

**B_sp_1011**  293 **FRDNTDNMYGLKAMLEGSEVDYAQVNDQVTFIDNHDMERFHTSNGDRRKL** 342

**P_sp_T16**  321 **FRDNTDNMYGLKAMLEGSEVxYAQVNDQVTFIDNHDMERFHTSNGDRRKL** 370

**P_sp_FSL_R5** 320 **FRDNTDNMYGLKSMLEGSATDYAQVEDQVTFIDNHDMERFHDSNANRRKL** 369

**B_sp_6.3.3**  327 **FRDNTSNMYALDSMINSTATDYNQVNDQVTFIDNHDMDRFKTSAVNNRRL** 376

**P_sp_P22**  329 **FRDGTDTMYGLDAMVAATGSDYAQVSDQVTFIDNHDMERFKTGSLSNRRL** 378

**H_larsenii**  324 **LRDFTDDWHGFKAMLDETAAEHDQVIDQVPFIDNHDMPRFTVEGGDTRNT** 373

**B_lichenifo** 327 **FRDNTSNMYALDSMLTATAADYNQVNDQVTFIDNHDMDRFKTSAVNNRRL** 376

**P_pabuli**  327 **FRDNTSNMYALDSMITGTAADYNQVNDQVTFIDNHDMDRFKTSAVNNRRL** 376

**B_sp_38-2**  320 **FRDNTDNMYGLKAMLEGSEVDYAQVNDQVTFIDNHDMERFHTSNGDRRKL** 369

**410 420 430 440 450**

**....|....|....|....|....|....|....|....|....|....|**

**B_sp_G825-6** 358 **DLGLALLLTSRGVPTIYYGTEIYMTGDGDPDNRKMMNTFDQSTVAYQIIQ** 407

**_B_firmus_l** 358 **DLGLAFLLTSRGVPTIYYGTEIYMTGDGDPDNRKMMNTFDQSTVAYQIIQ** 407

**_B_clarkii_** 361 **DIGLAFLLTSRGVPAIYYGTENYMTGKGDPGNRKMMESFDQTTTAYQVIQ** 410

**_B_agaradha** 340 **DIGLALMLTSRGVPTIYYGTEQYMEGDGDPGSRGMMASFDENTDAYKLIQ** 389

**B_sp_BL-31**  374 **DIGLALMLTSRGVPTIYYGTEQYMEGDGDPGSRAMMASFDENTDAYKLIQ** 423

**G_stearothe** 339 **DMALAVLLTSRGVPNIYYGTEQYMTGNGDPNNRKMMSSFNKNTRAYQVIQ** 388

**T_thermosul** 343 **EQALAFTLTSRGVPAIYYGTEQYMTGNGDPYNRAMMTSFNTSTTAYNVIK** 392

**B_sp_20RF**  365 **DIALAVLLTSRGVPTIYYGTEQYLTGGNDPDNRKPMNDFDRSTTSYQIIS** 414

**C_subterran** 370 **EQALAFTLTSRGVPAIYYGTEQYMTGNGDPYNRAMMTSFNTNTTAYNVIK** 419

**A_gottschal** 383 **EIGLAFLLTSRGVPTIYYGTEQYLTGNGDPYNRKPMSSFDQNTKAYKIIQ** 432

**B_obhensis**  365 **DIALAVLLTSRGVPTIYYGTEQYLTGGNDPENRKPMSDFDRTTNSYQIIS** 414

**B_cereus**  356 **DIALAVLLTSRGVPTIYYGTEQYLTGGNDPDNRKPMKTFDRSTNSYKITS** 405

**B_sp_1-1**  365 **DMALAVLLTSRGVPTIYYGTEQYVTGGNDPENRKPLKTFDRSTNSYQIIS** 414

**B_sp_BPED10** 365 **DMALAVLLTSRGVPTIYYGTEQYVTGGNDPENRKPLKTFDRSTNSYQIIS** 414

**B_brevis_CD** 354 **DIALAVLLTSRGVPTIYYGTEQYLTGGNDPDNRKPMKTFDRSTNSYKITS** 403

**B_sp_NR5_UP** 382 **DMALAVLLTSRGVPTIYYGTEQYVTGGNDPENRKPLKTFDRSTNSYQIIS** 431

**B_sp_G1-200** 365 **DMALAVLLTSRGVPTIYYGTEQYVTGGNDPENRKPLKTFDRSTNSYQIIS** 414

**B_sp_KC201**  387 **DMALAVLLTSRGVPTIYYGTEQYVTGGNDPENRKPLKTFDRSTNSYQIIS** 436

**P_macerans**  371 **EQALALTLTSRGVPAIYYGTEQYMTGDGDPNNRAMMTSFNTGTTAYKVIQ** 420

**B_firmus**  382 **DMALAVLLTSRGVPTIYYGTEQYVTGGNDPENRKPLKTFDRSTNSYQIIS** 431

**B_Circulans** 343 **EQALAFTLTSRGVPAIYYGTEQYMSGGTDPDNRARIPSFSTSTTAYQVIQ** 392

**B_sp_TS1-1**  382 **DMALAVLLTSRGVPTIYYGTEQYVTGGNDPENRKPLKTFDRSTNSYQIIS** 431

**B_sp_1011**  343 **EQALAFTLTSRGVPAIYYGSEQYMSGGNDPDNRARLPSFSTTTTAYQVIQ** 392

**P_sp_T16**  371 **EQALAFTLTSRGVPAIYYGSEQYMSGGNDPDNRARIPSFSTTTTAYQVIQ** 420

**P_sp_FSL_R5** 370 **EQALAFTLTSRGVPAIYYGTEQYMAGGNDPNNRARIPSFSTTTTAYQVIQ** 419

**B_sp_6.3.3**  377 **EQALAFTLTSRGVPAIYYGTEQYLTGNGDPDNRAKMPSFSKSTTAFNVIS** 426

**P_sp_P22**  379 **EQALAFTLTSRGVPAIYYGTEQYMTGGTDPDNRAMMPSFSTTTTAYKVSG** 428

**H_larsenii**  374 **DMALAVLLTSRGTPTVYYGTEQYLTGGNDPDNRKPMPSFDTTTTAYEVIQ** 423

**B_lichenifo** 377 **EQALAFTLTSRGVPAIYYGTEQYLTGNGDPDNRGKMPSFSKSTTAFNVIS** 426

**P_pabuli**  377 **EQALAFTLTSRGVPAIYYGTEQYLTGNGDPDNRAKMPSFSKTTTAFNVIS** 426

**B_sp_38-2**  370 **EQALAFTLTSRGVPAIYYGSEQYMSGGNDPDNRARIPSFSTTTTAYQVIQ** 419

**460 470 480 490 500**

**....|....|....|....|....|....|....|....|....|....|**

**B_sp_G825-6** 408 **RLSSLRQENRAIAYGDTTERWINEDVFIYERSFNGEYALIAVNRNLNRSY** 457

**_B_firmus_l** 408 **QLSSLRQENRAIAYGDTTERWINEDVFIYERSFNGEYALIAVNRSLNHSY** 457

**_B_clarkii_** 411 **KLAPLRQENKAVAYGSTKERWINDDVLIYERSFNGDYLLVAINKNVNQAY** 460

**_B_agaradha** 390 **KLAPLRKSNPAYGYGTTTERWINDDVLIYERHFGENYALIAINRNLNTSY** 439

**B_sp_BL-31**  424 **KLAPLRKSNPAYGYGTTTERWINDDVLIYERNFGENYALIAINRNLNTSY** 473

**G_stearothe** 389 **KLSSLRRNNPALAYGDTEQRWINGDVYVYERQFGKDVVLVAVNRSSSSNY** 438

**T_thermosul** 393 **KLAPLRKSNPAIAYGTTQQRWINNDVYIYERKFGNNVALVAINRNLSTSY** 442

**B_sp_20RF**  415 **SLASLRQSNPALEYGDTTERWINSDVYIYERKFGNNIVLTAVNSG-NTSY** 463

**C_subterran** 420 **KLAPLRKSNPAIAYGTQKQRWINNDVYIYERQFGKNVALIAINRNLSTSY** 469

**A_gottschal** 433 **KLAPLRKSNPALAYGTTQERWLNNDVIIYERKFGNNIVLVAINRNLSQSY** 482

**B_obhensis**  415 **TLASLRQNNPALGYGNTSERWINSDVYIYERSFGDSVVLTAVNSG-DTSY** 463

**B_cereus**  406 **KLASLRQRNSALGYGNTTERWINSDVYIYERKFGNSIVLTAVNSS-NRNQ** 454

**B_sp_1-1**  415 **KLASLRQTNSALGYGTTTERWLNEDIYIYERTFGNSIVLTAVNSS-NSNQ** 463

**B_sp_BPED10** 415 **KLASLRQTNSALGYGTTTERWLNEDIYIYERKFGNSIVLTAVNSS-NSNQ** 463

**B_brevis_CD** 404 **KLASLRQRNSALGYGNTTERWINSDVYIYERKFGNSIVLTAVNSS-NRNQ** 452

**B_sp_NR5_UP** 432 **KLASLRQTNSALGYGTTTERWLNEDIYIYERTFGNSIVLTAVNSS-NSNQ** 480

**B_sp_G1-200** 415 **KLASLRQTNSALGYGTTTERWLNEDIYIYERTFGNSIVLTAVNSS-NSNQ** 463

**B_sp_KC201**  437 **KLASLRQTNSALGYGTTTERWLNEDIYIYERTFGNSIVLTAVNSS-NSNQ** 485

**P_macerans**  421 **ALAPLRKSNPAIAYGTTTERWVNNDVLIIERKFGSSAALVAINRNSSAAY** 470

**B_firmus**  432 **KLASLRQTNSALGYGTTTERWLNEDIYIYERTFGNSIVLTAVNSS-NSNQ** 480

**B_Circulans** 393 **KLAPLRKCNPAIAYGSTQERWINNDVLIYERKFGSNVAVVAVNRNLNAPA** 442

**B_sp_TS1-1**  432 **KLASLRQTNSALGYGTTTERWLNEDIYIYERTFGNSIVLTAVNSS-NSNQ** 480

**B_sp_1011**  393 **KLAPLRKSNPAIAYGSTHERWINNDVIIYERKFGNNVAVVAINRNMNTPA** 442

**P_sp_T16**  421 **KLAPLRKSNPAIAYGSTQXRWINNDVIIYERKFGNNVAVVAINRNMNTPA** 470

**P_sp_FSL_R5** 420 **KLAPLRKSNPAIAYGTTQERWINNDVLIYERKFGNSAAVIAVNRNVNTPA** 469

**B_sp_6.3.3**  427 **KLAPLRKSNPAIAYGSTQQRWINNDVYVYERKFGKSVAVVAVNRNLSTPA** 476

**P_sp_P22**  429 **LLGPLRKSNPAIAYGTTQQRWINNDVYIYERKFGNNVAVVAVNRNLSTAA** 478

**H_larsenii**  424 **ELTALRSSNPALAYGDTQERWINSDVFIYEREFGDNVVLVAINRS-LDWY** 472

**B_lichenifo** 427 **KLAPLRKSNPAIAYGSTQQRWINNDVYIYERKFGKSVAVVAVNRNLTTPT** 476

**P_pabuli**  427 **KLAPLRKSNPAIAYGSTQQRWISNDVYVYERKFGKSVAVVAVNRNLSTPA** 476

**B_sp_38-2**  420 **KLAPLRKSNPAIAYGSTQERWINNDVIIYERKFGNNVAVVAINRNMNTPA** 469

**510 520 530 540 550**

**....|....|....|....|....|....|....|....|....|....|**

**B_sp_G825-6** 458 **QISSLVTDMPSQLYEDELSGLLDGQSITVAQDGSVQPFLLAPGEVSVWQY** 507

**_B_firmus_l** 458 **QISSLVTDMPSQLYEDELSGLLDGQSITVDQNGSIQPFLLAPGEVSVWQY** 507

**_B_clarkii_** 461 **TISGLLTEMPAQVYHDVLDSLLDGQSLAVKENGTVDSFLLGPGEVSVWQH** 510

**_B_agaradha** 440 **NIQGLQTEMPSNSYDDVLDGLLDGQSIVVDNKGGVNEFQMSPGEVSVWEF** 489

**B_sp_BL-31**  474 **NIQGLQTEMPSNSYDDVLDGLLDGQSIFVDNKGGVSGFQMSPGEVSVWEF** 523

**G_stearothe** 439 **SITGLFTALPAGTYTDQLGGLLDGNTIQVGSNGSVNAFDLGPGEVGVWAY** 488

**T_thermosul** 443 **NITGLYTALPAGTYTDVLGGLLNGNSISVASDGSVTPFTLSAGEVAVWQY** 492

**B_sp_20RF**  464 **SISNLNTSLPQGQYTDELHQLLDGNTITVNQNGSVNSFNLSPNGVSVWQF** 513

**C_subterran** 470 **YITGLYTALPAGTYSDVLGGLLNGNSITVSSNGSVTPFTLAPGEVAVWQY** 519

**A_gottschal** 483 **SITGLNTKLPEGYYYDELDGLLSGKSITVNPDGSVNQFIINPGEVSIWQF** 532

**B_obhensis**  464 **TINNLNTSLPQGQYTDELQQLLDGNEITVNSNGAVDSFQLSANGVSVWQI** 513

**B_cereus**  455 **TISNLNTSLPQGNYTDELQQLLDGNTITVNANGSANSFQLQANSVAVWQV** 504

**B_sp_1-1**  464 **TITNLNTSLPQGNYTDELQQRLDGNTITVNANGAVNSFQLRANSVAVWQV** 513

**B_sp_BPED10** 464 **TITNLNTSLPQGNYTDELQQRLDGNTITVNANGAVNSFQLRAHSVAVWQV** 513

**B_brevis_CD** 453 **TISNLNTSLPQGNYTDELQQLLDGNTITVNANGSANSPQLQANSVAVWQV** 502

**B_sp_NR5_UP** 481 **TITNLNTSLPQGNYTDELQQRLDGNTITVNANGAVNSFPLRANSVAVWQV** 530

**B_sp_G1-200** 464 **TITNLNTSLPQGNYTDELQQRLDGNTITVNANGAVNSFQLRANSVAVWQV** 513

**B_sp_KC201**  486 **TITNLNTSLPQGNYTDELQQRLDGNTITVNANGAVNSFQLRANSVAVWQV** 535

**P_macerans**  471 **PISGLLSSLPAGTYSDVLNGLLNGNSITVGSGGAVTNFTLAAGGTAVWQY** 520

**B_firmus**  481 **TITNLNTSLPQGNYTDELQQRLDGNTITVNANGAVNSFQLRANSAAVWQV** 530

**B_Circulans** 443 **SISGLVTSLPQGSYNDVLGGLLNGNTLSVGSGGAASNFTLAAGGTAVWQY** 492

**B_sp_TS1-1**  481 **TITNLNTSLPQGNYTDELQQRLDGNTITVNANGAVNSFQLRANSVAVWQV** 530

**B_sp_1011**  443 **SITGLVTSLPRGSYNDVLGGILNGNTLTVGAGGAASNFTLAPGGTAVWQY** 492

**P_sp_T16**  471 **SITGLVTSLPQGSYNDVLGGILNGNTLTVGAGGAASNFTLAPGGTAVWQY** 520

**P_sp_FSL_R5** 470 **SITGLATSLPAGNYADVLGGLLNGNSLTVSSGGAASNFTLAAGGTAVWQY** 519

**B_sp_6.3.3**  477 **NITGLSTSLPTGSYTDVLGGVLNGNNITSS-NGSVNSFTLAAGATAVWQY** 525

**P_sp_P22**  479 **SISGLVTSLPAGTYNDVLGGLLGGNSITAASGGAVGTFTLAAGGTAVWQY** 528

**H_larsenii**  473 **DVSGLVTSLPEGTYDDVLGGTLDGFSTTVNADGSIDTFSFGPQTVCVWEY** 522

**B_lichenifo** 477 **SITNLNTSLPSGTYTDVLGGVLNGNNITSS-GGNISSFTLAAGATAVWQY** 525

**P_pabuli**  477 **SIANLSTSLPTGNYTDVLGGALNGNNITST-NGNVSSFTLAAGATAVWQY** 525

**B_sp_38-2**  470 **SITGLVTSLPQGSYNDVLGGILNGNTLTVGAGGAASNFTLAPGGTAVWQY** 519

**560 570 580 590 600**

**....|....|....|....|....|....|....|....|....|....|**

**B_sp_G825-6** 508 **SNGQNVAPEIGQIGPPIGKPGDEVRIDGSGFGNSMGNVSFAGSTMN---V** 554

**_B_firmus_l** 508 **SNGQNVAPEIGQIGPPIGKPGDEVRIDGSGFGSSTGDVSFAGSTMN---V** 554

**_B_clarkii_** 511 **ISESGSAPVIGQVGPPMGKPGDAVKISGSGFGSEPGTVYFRDTKID---V** 557

**_B_agaradha** 490 **EAENVDKPSIGQVGPIIGEAGRTVTISGEGFGSSQGTVHFGSTSAE---I** 536

**B_sp_BL-31**  524 **EATNVDKPSIGQVGPIIGEAGRTVTISGEGFGSSQGTVHFGSTSAE---I** 570

**G_stearothe** 489 **SA-TESTPIIGHVGPMMGQVGHQVTIDGEGFGTNTGTVKFGTTAAN---V** 534

**T_thermosul** 493 **VS-SSNSPLIGHVGPTMTKAGQTITIDGRGFGTTSGQVLFGSTAGT---I** 538

**B_sp_20RF**  514 **TE-ETTSPLIGHVGPMMGKAGNTITINGEGFGDNEGNVLFNSSSSE---V** 559

**C_subterran** 520 **VS-TTNPPLIGHVGPTMTKAGQTITIDGRGFGTTAGQVLFGTTPGT---I** 565

**A_gottschal** 533 **AG-ETITPLIGQVGPIMGQVGNKVTISGVGFGDKKGTVNFGEIDAT---I** 578

**B_obhensis**  514 **TE-EHASPLIGHVGPMMGKHGNTVTITGEGFGDNEGSVLFDSDFSD---V** 559

**B_cereus**  505 **TK-ESTSPLIGHVGPMMGKTGNTVTVSGEGFGDKKGSVLFGSTSAE---I** 550

**B_sp_1-1**  514 **SN-PSTSPLIGQVGPMMGKAGNTITVSGEGFGDERGSVLFDSTSSE---I** 559

**B_sp_BPED10** 514 **SN-PSTSPLIGQVGPMMGKAGNTITVSGEGFGDERGSVLFDSTSSE---I** 559

**B_brevis_CD** 503 **TK-ESTSPLIGHVGPMIGKTGNTVTVSGEGFGDKKGSVLFGSTSAE---I** 548

**B_sp_NR5_UP** 531 **SN-PSTSPLIGQVGPMMGKAGNTITVSGQGFGDERGSVLFDSTSSE---I** 576

**B_sp_G1-200** 514 **SN-PSTSPLIGQVGPMMGKSGNTITVSGEGFGDERGSVLFDSTSSE---I** 559

**B_sp_KC201**  536 **SN-PSTSPLIGQVGPMMGKAGNTITVSGEGFGDERGSVLFDSTSSE---I** 581

**P_macerans**  521 **TA-PETSPAIGNVGPTMGQPGNIVTIDGRGFGGTAGTVYFGTTAVTGSGI** 569

**B_firmus**  531 **SN-PSTSPLIGQVGPMMGKAGNTITVSGEGFGDERGSVLFDSTSSE---I** 576

**B_Circulans** 493 **TA-ATATPTIGHVGPMMAKPGVTITIDGRGFGSSKGTVYFGTTAVSGADI** 541

**B_sp_TS1-1**  531 **SN-PSTSPLIGQVGPMMGKSGNTITVSGEGFGDERGSVLFDSTSSE---I** 576

**B_sp_1011**  493 **TT-DATTPIIGNVGPMMAKPGVTITIDGRGFGSGKGTVYFGTTAVTGADI** 541

**P_sp_T16**  521 **TT-DATAPIIGNVGPMMAKPGVTITIDGRGFGSGKGTVYFGTTAVTGADI** 569

**P_sp_FSL_R5** 520 **TT-ATTAPTIGHVGPMMAKPGATVTIDGRGFGASKGTVYFGTTAVTGANI** 568

**B_sp_6.3.3**  526 **TA-AETTPTIGHVGPVMGKPGNVVTIDGRGFGSTKGTVYFGTTAVTGAAI** 574

**P_sp_P22**  529 **TA-AATAPVVGHAGPVMGKPGNTVTIDGRGFGSSKGTVYFGSTAVTGTGI** 577

**H_larsenii**  523 **TG-TTTEPTLGHVGPTMGQPGHTVVLSGEGFGDTEGTVEFGTTAAS---I** 568

**B_lichenifo** 526 **TA-SETTPTIGHVGPVMGKPGNVVTIDGRGFGSAKGTVYFGTTAVTGSAI** 574

**P_pabuli**  526 **TT-SETTPTIGHVGPVMGKPGNVITISGRGFGSTKGTVYFGTSAVTGAAI** 574

**B_sp_38-2**  520 **TT-DATAPINGNVGPMMAKAGVTITIDGR-ASARQGTVYFGTTAVTGADI** 567

**610 620 630 640 650**

**....|....|....|....|....|....|....|....|....|....|**

**B_sp_G825-6** 555 **LSWNDETIIAELPVHNGGKNSITVTTNSGESSNGYP-FELLTGSQTSVRF** 603

**_B_firmus_l** 555 **LSWNDDTIIAELPEHNGGKNSVTVTTNSGESSNGYP-FELLTGLQTSVRF** 603

**_B_clarkii_** 558 **LTWDDETIVITLPETLGGKAQISVTNSDGVTSNGYD-FQLLTGKQESVRF** 606

**_B_agaradha** 537 **LSWNDTIITLTVPNNEAGYHDITVVTEDEQVSNAYE-FEVLTADQVTVRF** 585

**B_sp_BL-31**  571 **LSWNDTVITLTVPNNEAGYHDITVVTEDEQVSNAYE-FEVLTADQVTVRF** 619

**G_stearothe** 535 **VSWSNNQIVVAVPNVSPGKYNITVQSSSGQTSAAYDNFEVLTNDQVSVRF** 584

**T_thermosul** 539 **VSWDDTEVKVKVPSVTPGKYNISLKTSSGATSNTYNNINILTGNQICVRF** 588

**B_sp_20RF**  560 **ISWSNSKIEIKVPNVTAGHYDITVVSANDSNSNTYDRFEVLTGDQVTVRF** 609

**C_subterran** 566 **VSWEDTEVKVKVPALTPGKYNVTLKTASGITSNSYNNINVLTGNQVCVRF** 615

**A_gottschal** 579 **ISWTNSVIQIEIPSVPAGNYEITVSSEGGEKSNSYN-FEVLTNKQIPVRF** 627

**B_obhensis**  560 **LSWSDTKIEVSVPDVTAGHYDISVVNAGDSQSPTYDKFEVLTGDQVSIRF** 609

**B_cereus**  551 **VSWSNTEIQVKVPNVTAGHYNLSVVNATNTKSPAYEKFEVLSGNQVSVRF** 600

**B_sp_1-1**  560 **ISWSNTKISVKVPNVAGGYYDLSVVTAANIKSPTYKEFEVLSGNQVSVRF** 609

**B_sp_BPED10** 560 **ISWSNTEISVKVPNVAGGYYDLSVVTAANIKSPTYKEFEVLSGNQVSVRF** 609

**B_brevis_CD** 549 **VSWSNTEIQVKVPNVTAGHYNLSVVNATNTKSPAYEKFEVLSGNQVSVRF** 598

**B_sp_NR5_UP** 577 **ISWSNTEISVKVPNVAGGYYDLSVVTAANIKSPAYKEFEVLSGNQVSVRF** 626

**B_sp_G1-200** 560 **ISWSNTEISVKVPNVAGGYYDLSVVTAANLKSPTYKEFEVLSGNQVSVRF** 609

**B_sp_KC201**  582 **ISWSNTKISVKVPNVAGGYYDLSVVTAANIKSPTYKEFEVLSGNQVSVRF** 631

**P_macerans**  570 **VSWEDTQIKAVIPKVAAGKTGVSVKTSSGTASNTFKSFNVLTGDQVTVRF** 619

**B_firmus**  577 **ISWSNTEISVKVPNVAGGYYDLSVVTAANIKSPTYKEFEVLSGNQVSVRF** 626

**B_Circulans** 542 **TSWEDTQIKVKIPAVAGGNYNIKVANAAGTASNVYDNFEVLSGDQVSVRF** 591

**B_sp_TS1-1**  577 **ISWSNTEISVKVPNVAGGYYDLSVVTAANLKSPTYKEFEVLSGNQVRVRF** 626

**B_sp_1011**  542 **VAWEDTQIQVKIPAVPGGIYDIRVANAAGAASNIYDNFEVLTGDQVTVRF** 591

**P_sp_T16**  570 **VAWEDTQIQVKIPAVPGGIYDIRVANAAGAASNIYDNFEVLTGDQVTVRF** 619

**P_sp_FSL_R5** 569 **TAWEDTQIKVKIPAVAGGVYNIKIANSGGASSNVYDNFEVLTGDQVSVRF** 618

**B_sp_6.3.3**  575 **TSWEDTQIKVTIPSVAAGNYAVKVAANG-VNSNAYNHFTILTGDQVTVRF** 623

**P_sp_P22**  578 **VSWEDTQIKVTVPSVAAGSYGIKIRTSGAVDSNVYNGFNILTGNQVSVRF** 627

**H_larsenii**  569 **VSWSDTEIEATVPAVAGGYYDITVTDANGVQSDAFSGYEVLSGDQISARF** 618

**B_lichenifo** 575 **TSWEDTQIKVTIPPVAGGDYAVKVAANG-VNSNAYNDFTILSGDQVSVRF** 623

**P_pabuli**  575 **TSWEDTQIKVTIPAVAAGNYAVKVAANG-VNSNAYNNFTILTGDQVTVRF** 623

**B_sp_38-2**  568 **VAWEDTQIQVKILRVPGGIYDIRVANAAGAASNIYDNFEVLTGDQVTVRF** 617

**660 670 680 690 700**

**....|....|....|....|....|....|....|....|....|....|**

**B_sp_G825-6** 604 **VVNQAETSVGENLYLVGNVPELGSWDP-DKAIGPMFNQVLYSYPTWYYDV** 652

**_B_firmus_l** 604 **VVNQAETSVGENLYVVGDVPELGSWDP-DKAIGPMFNQVLYSYPTWYYDV** 652

**_B_clarkii_** 607 **VVDNAHTNYGENVYLVGNVPELGNWNP-ADAIGPMFNQVVYSYPTWYYDV** 655

**_B_agaradha** 586 **IIDNAETKLGENVFLVGNVHELGNWDP-EQSVGRFFNQIVYQYPTWYYDV** 634

**B_sp_BL-31**  620 **IIDNAETKLGENVFLVGNVHELGNWDP-EQSVGKFFNQIVYQYPTWYYDV** 668

**G_stearothe** 585 **VVNNATTNLGQNIYIVGNVYELGNWDT-SKAIGPMFNQVVYSYPTWYIDV** 633

**T_thermosul** 589 **VVNNASTVYGENVYLTGNVAELGNWDT-SKAIGPMFNQVVYQYPTWYYDV** 637

**B_sp_20RF**  610 **AVNNATTNLGTNLYIVGNVSELGNWDP-DQAIGPMFNQVMYQYPTWYYDI** 658

**C_subterran** 616 **VVNNASTVWGENVYLTGNVAELGNWDT-SKAIGPMFNQVVYQYPTWYYDV** 664

**A_gottschal** 628 **VVNNAYTSWGQNVYLVGNVHELGNWDP-NRAIGPFFNQVVYQYPTWYLDI** 676

**B_obhensis**  610 **AVNNATTSLGTNLYMVGNVNELGNWDP-DQAIGPMFNQVMYQYPTWYYDI** 658

**B_cereus**  601 **AVNNATTNSGTNVYIVGNVSELGNWDP-NKAIGPMFNQVMYKYPTWYYDI** 649

**B_sp_1-1**  610 **GVNNATTSPGTNLYIVGNVNELGNWDA-DKAIGPMFNQVMYQYPTWYYDI** 658

**B_sp_BPED10** 610 **GVNNATTSPGTNLYIVGNVSELGNWDA-DKAIGPMFNQVMYQYPTWYYDI** 658

**B_brevis_CD** 599 **AVNNATTNSGTNVYIVGNVSELGNWDP-NKAIGPMFNQVMYKYPTWYYDI** 647

**B_sp_NR5_UP** 627 **GVNNATTSPGTNLYIVGNVSELGNWDA-DKAIGPMFNQVMYQYPTWYYDI** 675

**B_sp_G1-200** 610 **GVNNATTSPGTNLYIVGNVSELGNWDA-DKAIGPMFNQVMYQYPTWYYDI** 658

**B_sp_KC201**  632 **GVNNATTSPGTNLYIVGNVNELGNWDA-DKAIGPMFNQVMYQYPTWYYDI** 680

**P_macerans**  620 **LVNQANTNYGTNVYLVGNAAELGSWDP-NKAIGPMYNQVIAKYPSWYYDV** 668

**B_firmus**  627 **GVNNATTSPGTNLYIVGNVSELGNWDA-DKAIGPMFNQVMYQYPTWYYDI** 675

**B_Circulans** 592 **VVNNATTALGQNVYLTGSVSELGNWDP-AKAIGPMYNQVVYQYPNWYYDV** 640

**B_sp_TS1-1**  627 **GVNNATTSPGTNLYIVGNVSELGNWYA-DKAIGPMFNQLMFQYPTWYYDI** 675

**B_sp_1011**  592 **VINNATTALGQNVFLTGNVSELGNWDP-NNAIGPMYNQVVYQYPTWYYDV** 640

**P_sp_T16**  620 **VINNATTALGQNVFLTGNVSELGNWDP-NNAIGPMYNQVVYQYPTWYYDV** 668

**P_sp_FSL_R5** 619 **VINNATTALGQNVYLSGSVSELGNWDP-AKAIGPLYNQVIYQYPTWYYDV** 667

**B_sp_6.3.3**  624 **VINNASTTLGQNIYLTGNVAELGNWSTGSTAIGPAFNQVIHQYPTWYYDV** 673

**P_sp_P22**  628 **VVQNATTALGENVYLSGGVSELGNWAP-AAAIGPIFNKILYAYPTWYYDV** 676

**H_larsenii**  619 **VVNDATTDMGENVYVVGNVHELGNWDT-DRAVGPFFNQVVHEYPNWYYDV** 667

**B_lichenifo** 624 **VINNATTALGENIYLTGNVSELGNWTTGAASIGPAFNQVIHAYPTWYYDV** 673

**P_pabuli**  624 **VINNASTTLGQNIYLTGNVAELGNWSTGATAIGPAFNQVIHAYPTWYYDV** 673

**B_sp_38-2**  618 **VINNATTALGQNVFLTGNVSELGNWDP-NNAIGPMYNQVVYQYPTWYYDV** 666

**710 720 730 740**

**....|....|....|....|....|....|....|....|....|..**

**B_sp_G825-6** 653 **SVPANQDIEYKYIMKDQNGNVSWESGGNHIYRTPENSTGIVEVNYNQ** 699

**_B_firmus_l** 653 **SVPANQDIEYKYIMKDQNGNVSWESGNNHIYRTPENSTGIVEVNFNQ** 699

**_B_clarkii_** 656 **SVPADTALEFKFIIVDGNGNVTWESGGNHNYRVTSGSTDTVRVSFRR** 702

**_B_agaradha** 635 **NVPANTDLEFKFIKIDQDNNVTWQSGANQTYSSPESGTGIIRVDW--** 679

**B_sp_BL-31**  669 **NVPANTDLEFKFIKIDQDNNVTWQSGANQTYSSPESGTGIIRVDW--** 713

**G_stearothe** 634 **SVPEGKTIEFKFIKKDSQGNVTWESGSNHVYTTPTNTTGKIIVDWQN** 680

**T_thermosul** 638 **SVPAGTTIQFKFIKKNG-NTITWEGGSNHTYTVPSSSTGTVIVNWQQ** 683

**B_sp_20RF**  659 **SVPAEENLEYKFIKKDGNGNVVWESGDNHTYTSPPAGTDTVIVDWQ-** 704

**C_subterran** 665 **SVPAGTTIEFKFIKKNG-STVTWEGGYNHVYTTPTSGTATVIVNWQN** 710

**A_gottschal** 677 **SVPADTTLEFKFIKIDESGNVIWQSGLNRVYTTPEKGTDTIYFEW--** 721

**B_obhensis**  659 **SVPAEENLEYKFIKKDSSGNVVWESGNNHTYTTPATGTDTVLVDWQ-** 704

**B_cereus**  650 **SVPAGKNLEYKYIKKDQNGNVTWQSGNNRTYTSPATGTDTVISNW--** 694

**B_sp_1-1**  659 **SVPAGKNLEYKYIKKDQNGNVVWQSGNNRTYTSPTTGTDTVMINW--** 703

**B_sp_BPED10** 659 **SVPAGKNLEYKYIKKDQNGNVVWQSGNNRTYTSPTTGTDTVMINW--** 703

**B_brevis_CD** 648 **SVPAGKNLEYKYIKKDHNGNVTWQSGNNRTYTSPATGTDTVISNW--** 692

**B_sp_NR5_UP** 676 **SVPAGKNLEYKYIKKDQNGNVVWQSGNNRTYTSPTTGTDTVMINW--** 720

**B_sp_G1-200** 659 **SVPAGKNLEYKYIKKDQNGNVVWQSGNNRTYTSPTTGTDTVMINW--** 703

**B_sp_KC201**  681 **SVPAGKNLEYKYIKKDQNGNVVWQSGNNRTYTSPTTGTDTVMINW--** 725

**P_macerans**  669 **SVPAGTKLDFKFIKKGG-GTVTWEGGGNHTYTTPASGVGTVTVDWQN** 714

**B_firmus**  676 **SVPAGKNLEYKYIKKDQNGNVVWQSGNNRTYTSPTTGTDTVMID---** 719

**B_Circulans** 641 **SVPAGKTIEFKFLKKQG-STVTWEGGSNHTFTAPSSGTATINVNWQP** 686

**B_sp_TS1-1**  676 **SVPAGKNLEYKYIKKDQNGNVVWQSGNNRTYTSPTTGTDTVMINW--** 720

**B_sp_1011**  641 **SVPAGQTIEFKFLKKQG-STVTWEGGANRTFTTPTSGTATVNVNWQP** 686

**P_sp_T16**  669 **SVPAGQTIEFKFLKKQG-STVTWEGGANRTFTTPTSGTATMNVNW--** 712

**P_sp_FSL_R5** 668 **SVPAGKTIEFKFLKKQG-STVTWEGGSNHSFTAPASGTATVNVNWQP** 713

**B_sp_6.3.3**  674 **SVPAGKELEFKFFKKNG-STITWEGGSNHKFTTPASGTATVTVNWQ-** 718

**P_sp_P22**  677 **SVPAGTALQFKFFKKNG-TAVTWEGGSNHTFTTPATGTATVVVNWQ-** 721

**H_larsenii**  668 **NLPAGTDIEFKFVKIADDGTVTWESGSNRTYTTPTDSTGEYTGTWK-** 713

**B_lichenifo** 674 **SVPAGKQLEFKFFKKNG-ATITWEGGSNHTFTTPTSGTATVTINWQ-** 718

**P_pabuli**  674 **SVPAGKQLEFKFFKKNG-ATITWEGGSNHTFTTPASGTATVNVNWQ-** 718

**B_sp_38-2**  667 **SVPAGQTIEFKFLKKQG-STVTWEGGANRTFTTPTSGTATVNVNWQP** 712
